# CD163 and Tim-4 identify resident intestinal macrophages that are spatially regulated by TGF-β

**DOI:** 10.1084/jem.20240801

**Published:** 2026-04-21

**Authors:** Vignesh Jayaraman, Ian E. Prise, Verena Kästele, Sabrina Tamburrano, Kelly Wemyss, Hayley M. Bridgeman, Rufus H. Daw, Patrick Strangward, Alfie Sanderson, Sheena M. Cruickshank, Joanne E. Konkel, Christine Chew, Chengcan Yao, C.J. Anderson, Josef Priller, Barry McColl, David A.D. Munro, Liesbet Martens, Charlotte L. Scott, Martin Guilliams, Antony D. Adamson, John R. Grainger, Tovah N. Shaw

**Affiliations:** 1 Institute of Immunology and Infection Research, School of Biological Sciences, University of Edinburgh, Edinburgh, UK; 2 Lydia Becker Institute of Immunology and Inflammation Research, Faculty of Biology, Medicine and Health, The University of Manchester, Manchester, UK; 3 Centre for Inflammation Research, Institute for Regeneration and Repair, The University of Edinburgh, Edinburgh, UK; 4 UK Dementia Research Institute, University of Edinburgh, Edinburgh, UK; 5 Department of Psychiatry & Psychotherapy, TUM University Hospital, Technical University of Munich, and DZPG, Munich, Germany; 6 Laboratory of Molecular Psychiatry, Charité - Universitätsmedizin Berlin and DZNE, Berlin, Germany; 7 Laboratory of Myeloid Cell Biology in Tissue Damage and Inflammation, VIB-UGent Center for Inflammation Research, Ghent, Belgium; 8 Department of Biomedical Molecular Biology, Faculty of Sciences, Ghent University, Ghent, Belgium; 9 Laboratory of Myeloid Cell Biology in Tissue Homeostasis and Regeneration, VIB-UGent Center for Inflammation Research, Ghent, Belgium; 10 Genome Editing Unit, Faculty of Biology, Medicine and Health, Manchester Academic Health Science Centre, The University of Manchester, Manchester, UK

## Abstract

Macrophages localize in sub-tissular niches associated with their ontogeny and activity. In the intestine, a paradigm has emerged that long-lived macrophages are present in the muscular layer, while highly monocyte-replenished populations are found in the lamina propria (LP). Whether long-lived macrophages are restricted in such a simplified manner has not been well explored. Moreover, the impact of specific gut-associated factors on macrophage identity across intestinal tissue layers is unknown. We generated scRNA-seq data from WT and *Ccr2*^−/−^ mice to identify phenotypic features of long-lived macrophage populations in distinct intestinal layers and identified CD163 as a marker to distinguish submucosal/muscularis (S/M) from LP macrophages. Challenging the emerging paradigm, long-lived macrophages were found in the LP and S/M, with distinct transcriptomes and responsiveness to proinflammatory stimuli. Employing transgenic mice, we demonstrate a critical role for TGF-β signalling in maintaining the identity of long-lived LP but not S/M macrophages and that macrophage-derived TGF-β1 is required to instruct intestinal macrophage identity after development.

## Teaser

Jayaraman et al. challenge the prevailing paradigm that long-lived gut macrophages are restricted to the muscularis, identifying their presence in the lamina propria and using CD163 to distinguish these functionally distinct populations. Deletion of TGF-β signalling molecules demonstrates distinct sub-tissular dependencies of gut macrophages on TGF-β and that macrophages themselves are the key TGF-β–producing population.

## Graphical Abstract

**Figure:**
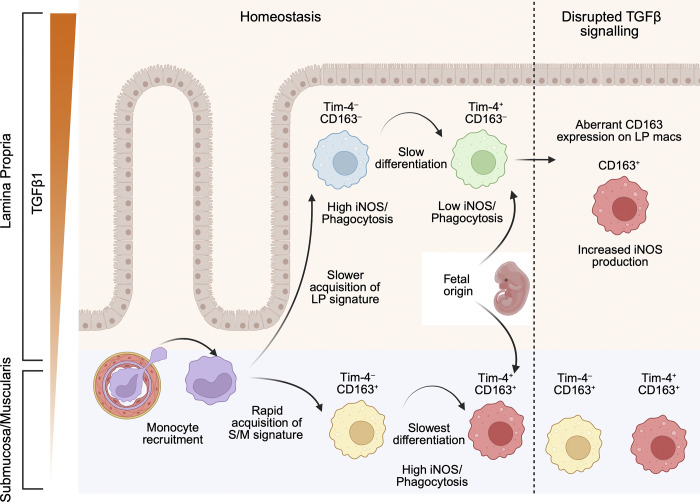

## Introduction

Transcriptional and functional heterogeneity of resident tissue macrophages (RTMs) has been described at the level of ontogeny (Ginhoux et al., 2010; Schulz et al., 2012; Sheng et al., 2015; van de Laar et al., 2016), tissue (Gautier et al., 2012; Lavin et al., 2014), and increasingly sub-tissular niche (Chakarov et al., 2019; Moura Silva et al., 2021; Schyns et al., 2019; Sierro et al., 2017; Van Hove et al., 2019). The implications of this extraordinary level of heterogeneity are in the process of being revealed but to date include the findings that RTMs fulfil functions tailored to supporting the tissue they reside in (Dranoff et al., 1994; Kohyama et al., 2009; Paolicelli et al., 2011) and the cells they are near to (He et al., 2016; Matheis et al., 2020). In turn, the adjacent cells in the spatial niche are thought to maintain and instruct the RTM in its function (Buechler et al., 2019; Guilliams et al., 2020; Muller et al., 2014). While several studies have begun to identify relationships between RTM location and function in the lung (Chakarov et al., 2019; Gibbings et al., 2017; Schyns et al., 2019), liver (Guilliams et al., 2022), and brain (Van Hove et al., 2019), the small intestine remains a site where these relationships are still relatively poorly understood. This is surprising given the diverse environments intestinal RTMs can be found in. For example, the intestine can be broadly divided into four layers: mucosa (including the lamina propria [LP]), submucosa, muscularis propria, and serosa (Arroyo Portilla et al., 2020; Gabanyi et al., 2016; Hume et al., 1984; Nagashima et al., 1996). Increasing the complexity, many macrophages in sub-tissular environments are found next to sub-tissular structures, such as nerves (De Schepper et al., 2018; Gabanyi et al., 2016; Muller et al., 2014) and blood vessels (De Schepper et al., 2018; Honda et al., 2020; Moura Silva et al., 2021).

As well as identifying macrophages in physically distinct compartments, intestinal macrophages with different levels of dependency on monocyte replacement have been described (Bujko et al., 2018; De Schepper et al., 2018; Shaw et al., 2018). In particular, we identified T cell immunoglobulin and mucin domain 4 (Tim-4) as a marker of intestinal macrophages with low levels of monocyte replenishment (Shaw et al., 2018). Prior to this, it was thought that all intestinal macrophages were continuously and rapidly replenished from monocytes, largely due to the tonic inflammation in the intestine driven by the high commensal microbial burden (Bain et al., 2014; Ginhoux and Jung, 2014). While there is now agreement on the presence of a long-lived intestinal macrophage population (Hegarty et al., 2023; Viola and Boeckxstaens, 2021), there is not yet consensus on the best markers to identify them, nor on the sub-tissular location of these cells. Indeed, the emerging paradigm is that the LP macrophages present in the tonically inflamed environment beneath the intestinal epithelium are constantly replenished from monocytes, while long-lived macrophages are present in the deeper tissues, such as the submucosa and muscularis (S/M) (Hegarty et al., 2023; Viola and Boeckxstaens, 2021). Resulting from the lack of consensus on markers to identify these long-lived macrophages and knowledge about where they are located, the functions of, and factors regulating, long-lived macrophages in distinct sub-tissular layers of the intestine have not been explored.

## Results

**Figure S1.**
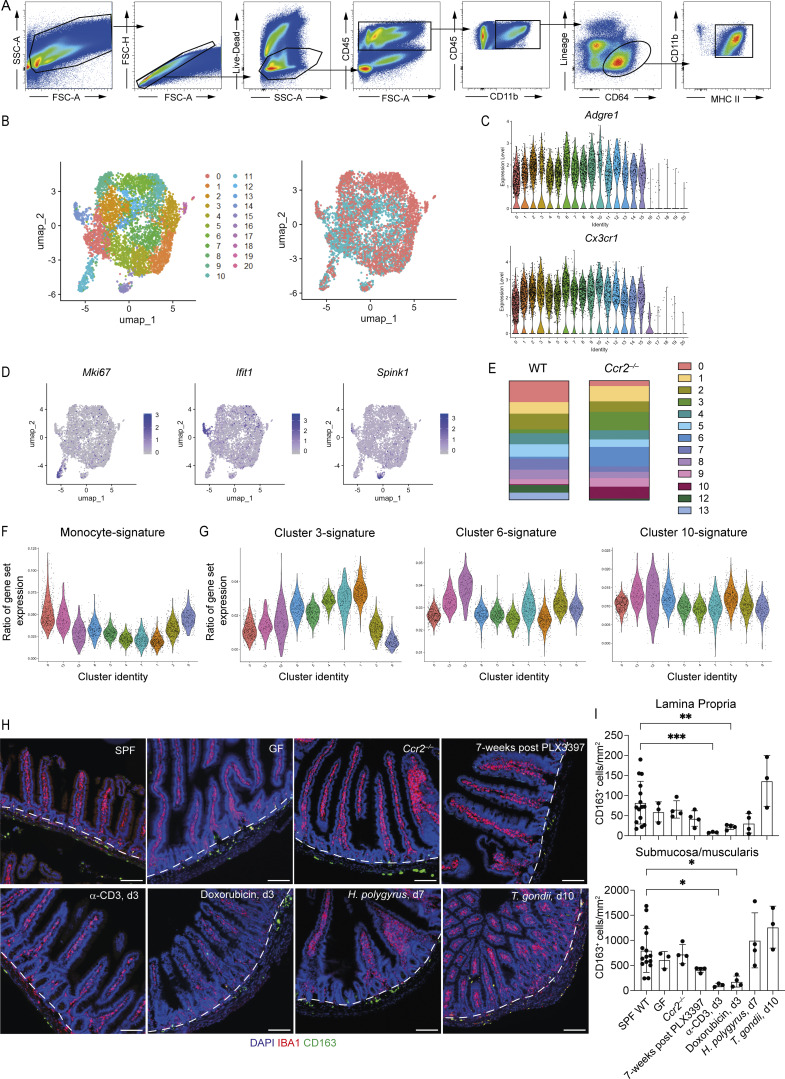
**Identification and curation of scRNA-seq clusters from WT and *Ccr2*** ^**−/−**^ **mice. (A)** Flow cytometry gating strategy for identification of small intestinal macrophages from a WT C57BL/6 mouse. **(B)** UMAP plots of scRNA-seq data from 5,639 live CD45^+^Lin^−^CD11b^+^MHCII^+^CD64^+^ cells from the small intestine of two WT and four *Ccr2*^−/−^ mice. Left: Colors denote individual cells assigned to the same cluster. Right: Colors denote cells derived from WT (blue) or *Ccr2*^−/−^ (red) mice. **(C)** Violin plots showing expression of *Adgre1* (F4/80) and *Cx3cr1* (CX3CR1) in each cluster. **(D)** UMAP plots showing expression levels of *Mki67, Ifit1*, and *Spink1*. **(E)** Proportions of each macrophage cluster by mouse genotype. **(F)** Violin plots showing the similarity score of each cluster against ImmGen datasets for monocytes. **(G)** Violin plots showing the similarity score of excluded clusters 3, 6, and 10 against each of the other clusters. **(H)** Representative immunofluorescence images of small intestine section from mice subject to a variety of infectious (*H*. *polygyrus*; *T. gondii*), inflammatory (α-CD3; doxorubicin), or non-homeostatic conditions (*Ccr2*^−/−^, germ-free mice, after PLX3397-mediated depletion). DAPI (blue), IBA1 (red), and CD163 (green). Dashed line demarcates the boundary between the S/M, and LP. Scale bar = 100 μm. **(I)** Quantification of CD163-expressing cells in the LP (upper) and S/M (lower) of the small intestine from GF, *Ccr2*^−/−^, and WT mice 7 wk after treatment with PLX3397 diet, 3 day after treatment with α-CD3, 3 day after treatment with doxorubicin, 7 day after infection with *H. polygyrus*, or 10 days after infection with *T. gondii*. Data for the SPF group are combined from the uninfected, untreated, and WT controls from all other groups. Data from *n* = 3–15 per group. Error bars show mean ± SD. Statistical comparisons were performed with a Brown–Forsythe and Welch ANOVA test for parametric data and a Kruskal–Wallis test with Dunn’s multiple comparison test for nonparametric data. *, P ≤ 0.05; **, P ≤ 0.01; ***, P ≤ 0.001. UMAP, uniform manifold approximation and projection.

To identify distinct populations of RTMs, we began by utilizing single-cell RNA sequencing (scRNA-seq) of live CD45^+^Lin^–^CD11b^+^MHCII^+^CD64^+^ cells (Fig. S1 A) from the small intestine of healthy WT and *Ccr2*^−/−^ mice (Boring et al., 1997). *Ccr2*^−/−^ mice have a paucity of recruited circulating monocytes (Serbina and Pamer, 2006), and we have previously shown that they are enriched for intestinal long-lived macrophages (Shaw et al., 2018). Comparison of the intestinal macrophage populations in these two strains was, therefore, used to help understand the relationship between macrophage populations and their ontogeny.

**Figure 1.**
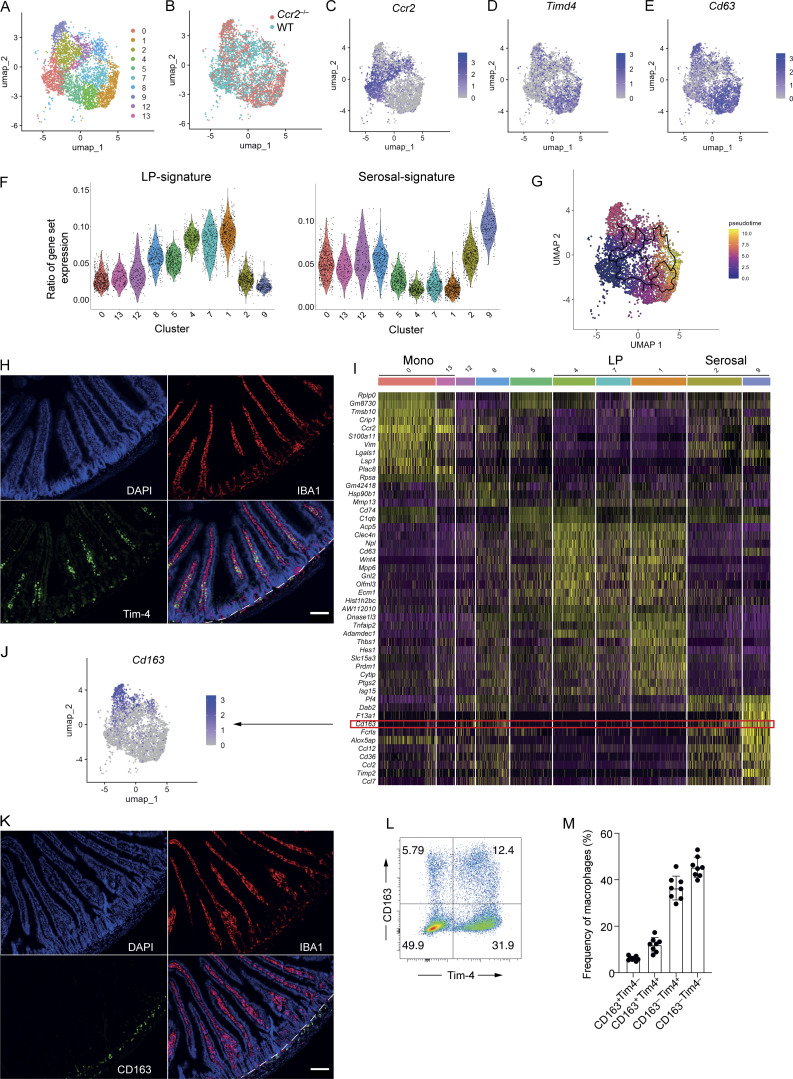
**Tim-4 and CD163 expression identify small intestinal macrophages in different sub-tissular locations. (A)** Uniform manifold approximation and projection (UMAP) plot of scRNA-seq data from 3,864 live CD45^+^Lin^−^CD11b^+^MHCII^+^CD64^+^ cells from the small intestine of two WT C57BL/6 and four *Ccr2*^−/−^ mice. Colors denote individual cells assigned to the same cluster. **(B)** UMAP plot showing the composition of clusters as derived from WT (blue) or *Ccr2*^−/−^ (red) mice. **(C)** UMAP plot showing expression level of *Ccr2*. **(D)** UMAP plot showing expression level of *Timd4*. **(E)** UMAP plot showing expression level of *Cd63.***(F)** Violin plots showing the similarity score of each cluster against ImmGen datasets for left, LP macrophages; right, serosal macrophages. **(G)** UMAP visualization of combined WT and *Ccr2*^−/−^-derived small intestinal macrophages showing their differentiation trajectory from *Ccr2*^+^ macrophages, colored by pseudotime as computed in Monocle 3. **(H)** Representative immunofluorescence image of small intestine section from WT mice. DAPI (blue), IBA1 (red), and Tim-4 (green). Dashed line demarcates the boundary between the LP (above the line) and S/M (below the line). **(I)** Heatmap showing the top DEGs for all small intestinal macrophage populations identified in Fig. 1 A, with *Cd163* highlighted. **(J)** UMAP plot showing expression level of *Cd163.***(K)** Representative immunofluorescence image of small intestine section from WT mice. DAPI (blue), IBA1 (red), and CD163 (green). Scale bar = 100 μm (H and K). Dashed line demarcates the boundary between the LP (above the line) and S/M (below the line). **(L)** Expression of CD163 and Tim-4 on small intestinal live CD45^+^Lin^–^CD11b^+^MHCII^+^CD64^+^ macrophages assessed by flow cytometry. Numbers denote the percentages of cells within the gate. **(M)** Frequency of macrophages expressing Tim-4 and/or CD163 in the small intestine. Error bars show mean ± SD. Data (*n* = 8 per group) are pooled from eight independent experiments.

Following automated clustering combined with manual assessment of gene expression profiles, we initially clustered 5,639 cells into 21 clusters that were interpretable as biologically meaningful (Fig. S1 B). All the clusters were assessed for expression of the intestinal macrophage markers *Adgre1* (F4/80) and *Cx3cr1* (CX3CR1) (Fig. S1 C). Given our intention to understand macrophage subsets and ontogeny in health, we excluded clusters that did not express high levels of *Adgre1* and *Cx3cr1* from further analysis (thus clusters 16–20, which were also the smallest, each comprising <50 cells, were excluded) (Fig. S1 C). We further excluded clusters that were likely defined by activation/functional states within macrophage subsets rather than subsets in their own right. These were cluster 11—proliferating macrophages (*Mki67*), cluster 14—IFN-activated macrophages (*Ifit1*), and cluster 15—macrophages that were likely associated with epithelial cells through doublet formation/phagocytosis (*Spink1*) (Fig. S1 D). Finally, we excluded clusters 3, 6, and 10, as these cells were largely absent from WT guts (<4% of WT macrophages) and found mostly only in *Ccr2*^−/−^ guts (Fig. S1 E), suggesting that they only developed in response to disruption of homeostatic macrophage differentiation. Thus, the final scRNA-seq map was generated, containing 10 clusters (Fig. 1 A). Each of these clusters was represented in WT and *Ccr2*^−/−^ mice, though the proportions of each cluster varied by genotype (Fig. S1 E).

We began by analyzing the scRNA-seq data to predict the ontogeny of the macrophage clusters. To this end, we compared clusters of different macrophage subsets between WT and *Ccr2*^−/−^ mice. Cluster 0 was most distinct from other populations in being almost completely comprised of WT macrophages, suggesting that this population is highly dependent on the constant recruitment of monocytes for its generation (Fig. 1 B and Fig. S1 E). *Ccr2* was enriched in this cluster and in clusters 8, 12, and 13 (Fig. 1 C), implying that they also had a strong dependency on monocytes. Contrasting this, clusters 1, 2, 4, and 9 were similarly represented in WT and *Ccr2*^−/−^ animals (Fig. 1 B and Fig. S1 E), suggesting that these macrophage populations are the least dependent on monocyte recruitment and were, therefore, more long-lived. Supporting this idea, these clusters were enriched for *Timd4* expression (Fig. 1 D), a marker shown by us and others to identify macrophages with lower dependency on monocyte replenishment (Dick et al., 2022; Shaw et al., 2018). Similarly, *Cd63*, also identified as a marker of intestinal macrophages with low dependency on monocyte replenishment (De Schepper et al., 2018), was enriched in clusters 1, 2, 4, and 9 (Fig. 1 E).

In addition to identifying clusters based on their inferred recent development from monocytes, we sought to identify their likely sub-tissular location by comparing their gene signatures with those of the signature of “LP” and “serosal” macrophages on the ImmGen database (Heng and Painter, 2008), with serosal macrophages likely to also include muscularis macrophages (Koscsó et al., 2015). Clusters 1, 4, and 7 scored most highly for “LP macrophage”–associated genes and clusters 2 and 9 for “serosal macrophage”–associated genes (Fig. 1 F). Clusters 8 and 5 had an intermediate LP score, suggesting they may be in the process of transitioning into mature LP macrophages. In line with their putative recent entry into the tissue, clusters 0, 13, and 12, enriched for *Ccr2,* were not enriched for either the LP or serosal macrophage gene signature (Fig. 1 F). As expected, and in line with our previous results (Shaw et al., 2018), expression of monocyte-associated genes by any of the clusters was weak, though *Ccr2*-enriched clusters, 0 and 13, scored more highly than the rest (Fig. S1 F). Interestingly, of the clusters that were present in WT and *Ccr2*^*−/−*^ mice and enriched for expression of long-lived, self-maintaining RTM genes (De Schepper et al., 2018; Shaw et al., 2018), *Timd4* (Fig. 1 D) and *Cd63* (Fig. 1 E), clusters 2 and 9 were found to score highly for the serosal macrophage signature and 1 and 4 for the LP macrophage signal (Fig. 1 F), suggesting that long-lived macrophages could be found in both sub-tissular locations. These results would, therefore, challenge the current established understanding that long-lived intestinal RTMs are located only within the S/M (Bain and Schridde, 2018; De Schepper et al., 2018; Hegarty et al., 2023; Viola and Boeckxstaens, 2021; Yip et al., 2021).

Separate analysis of earlier excluded clusters found only in *Ccr2*^−/−^ mice (3, 6, and 10) using a similar approach showed cluster 3 to express similar genes as clusters 1, 4, and 7, while cluster 6 expressed similar genes as clusters 12 and 13 (Fig. S1 G), supporting our hypothesis that these clusters represent populations of macrophages usually present in WT mice that have experienced a disrupted differentiation process in *Ccr2*^−/−^ mice, such that they develop a different activation state. Cluster 10 showed no similarity to any particular cluster, suggesting its unique development in *Ccr2*^−/−^ mice (Fig. S1 G).

To better explore the differentiation trajectory of gut macrophages from their *Ccr*2^+^ status upon entry into the tissue, we undertook trajectory analysis using Monocle 3 (Trapnell et al., 2014). Using cluster 0, enriched for *Ccr2* expression, as the seed cluster, our trajectory analysis suggests that *Ccr2*^+^ macrophages can differentiate along several pathways, diverging into terminally differentiated macrophages in cluster 2 or progressing through alternate pathways with several routes to terminal differentiation in cluster 4. According to pseudotime analysis, differentiation into cluster 2 is predicted to happen more rapidly than differentiation into cluster 4. Cluster 1, highly enriched for expression of *Timd4*, is predicted to take the longest time to develop, with several pathways converging on this cluster (Fig. 1 G). Together, these data suggest that there may be multiple developmental pathways to becoming a long-lived macrophage in the gut, and that it is possible that differentiation into a serosal macrophage occurs more rapidly than differentiation into an LP macrophage.

To validate our sequencing results, we assessed the spatial location of RTMs in the small intestine using immunofluorescence, staining for Tim-4 as a marker of long-lived macrophages (Shaw et al., 2018), and found that, in agreement with our sequencing data, Tim-4^+^ macrophages were located in the villi of the LP, with a second, albeit smaller population, also located in the S/M (Fig. 1 H). To identify markers that would distinguish between Tim-4^+^ macrophages in these different locations, we assessed the top differentially expressed genes (DEGs) for each cluster and identified *Cd163* as a highly specific candidate marker of S/M macrophages, enriched in clusters 2 and 9 (Fig. 1, I and J). Immunofluorescence imaging, as predicted by our sequencing, showed that CD163-expressing macrophages were almost entirely restricted in their location to the S/M of the small intestine (Fig. 1 K). We assessed whether restriction of CD163^+^ macrophages to the S/M of the small intestine was only a feature of health or was maintained following perturbation to the intestinal environment. Localization of CD163^+^ cells was unchanged: (1) during type 1 (*Toxoplasma gondii*) and type 2 (*Heligmosomoides polygyrus bakeri*) intestinal infections; (2) in response to epithelial cell death induced by the chemotherapy doxorubicin or to T cell–induced epithelial damage following α-CD3 treatment; (3) in *Ccr2*^−/−^ mice or where macrophages had been previously depleted and allowed to repopulate (7-wk after PLX3397); and (4) in germ-free animals lacking commensal microbiota. There was, however, a decrease in the few CD163^+^ cells found in the LP in response to α-CD3 or doxorubicin treatment, and an overall decrease in numbers of CD163^+^ cells in the S/M in these same settings (Fig. S1, H and I). Thus, CD163 can be used as a reliable marker to distinguish LP versus S/M macrophages across a variety of contexts.

Furthermore, assessment of small intestinal macrophages for CD163 and Tim-4 protein expression showed that they could be separated into four populations: CD163^−^Tim-4^+^, CD163^+^Tim-4^−^, CD163^+^Tim-4^+^, and CD163^−^Tim-4^−^ (Fig. 1, L and M). These data corroborate, at protein level, that two populations of Tim-4^+^ macrophages can be identified (CD163^−^ and CD163^+^), likely present in the LP and S/M, respectively. Taken together, these results reveal that Tim-4 may be a marker of longevity, independent of location, while differential expression of CD163 represents an unappreciated marker reflective of sub-tissular location.

**Figure S2.**
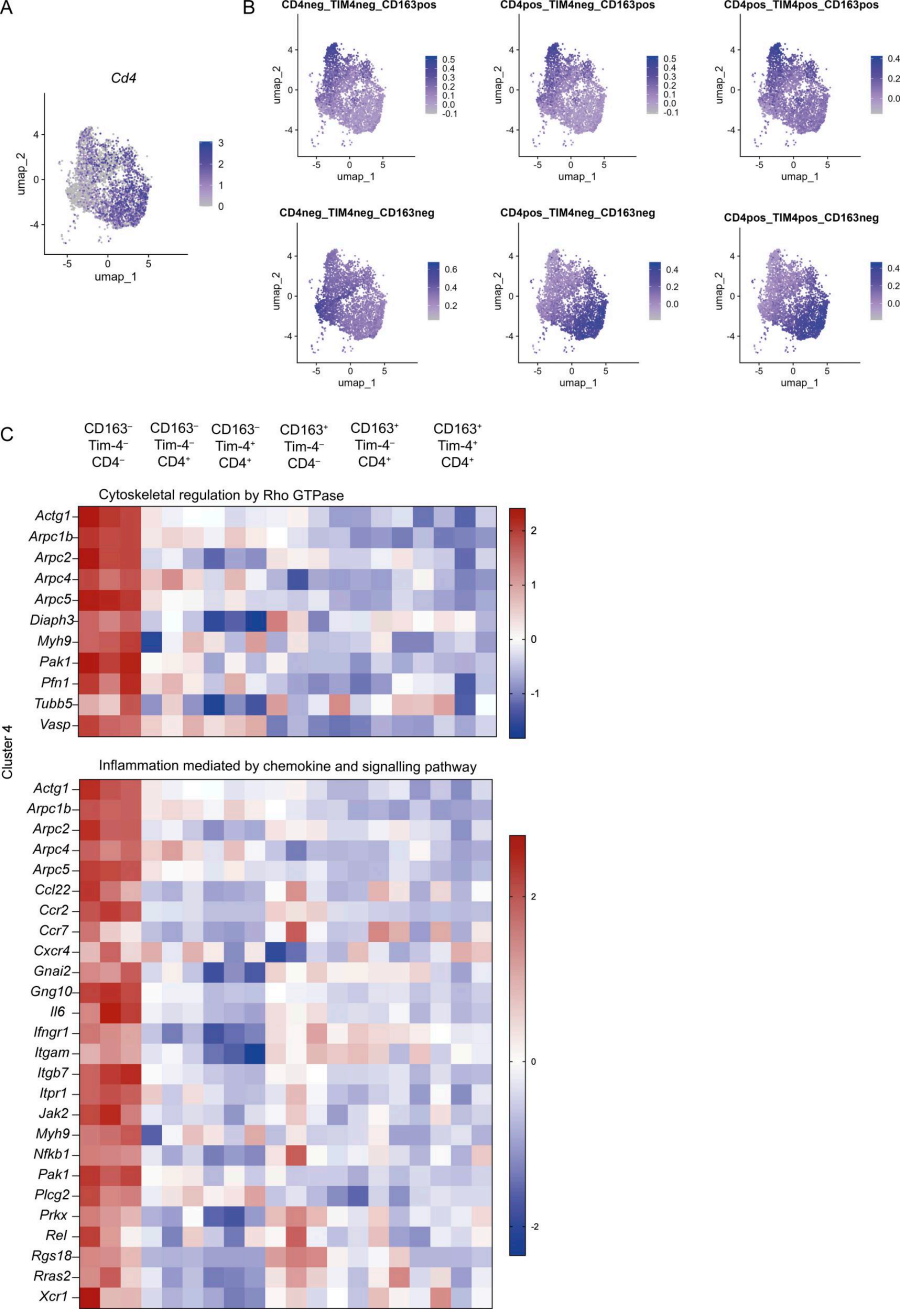
**Gene set pathway analysis for cluster 4. (A)** UMAP plot showing expression levels of *Cd4*. **(B)** UMAP plots showing cells expressing genes from sorted CD163^−^ and CD163^+^ subsets of Tim-4^−^CD4^−^, Tim-4^−^CD4^+^, and Tim-4^+^CD4^+^ macrophages of the small intestine of WT mice overlaid onto scRNA-seq data from live CD45^+^Lin^−^CD11b^+^MHCII^+^CD64^+^ cells from the small intestine of WT and *Ccr2*^−/−^ mice. **(C)** Heatmaps showing expression profiles of genes in the top two pathways identified by PANTHER pathway analysis for cluster 4. **(B and C)** CD163^−^ and CD163^+^ subsets of Tim-4^−^CD4^−^, Tim-4^−^CD4^+^, and Tim-4^+^CD4^+^ macrophage of the small intestine were isolated by FACS from five pooled 8–10-wk-old C57BL/6 WT mice, from at least three independent sorts, for bulk RNA-seq. UMAP, uniform manifold approximation and projection.

**Figure 2.**
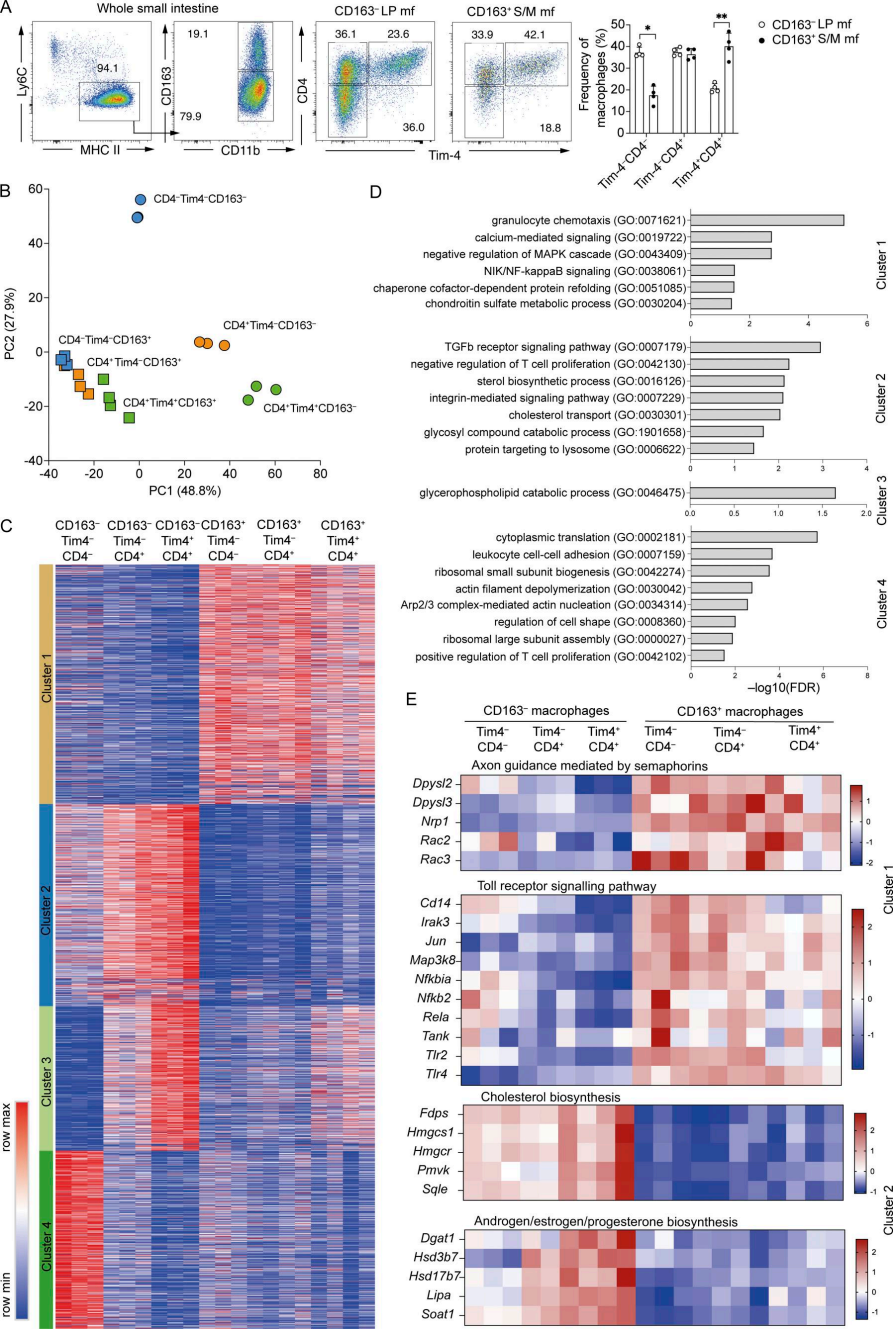
**CD4, Tim-4, and CD163 identify transcriptionally distinct macrophages in the small intestine. (A)** Left: Representative flow cytometry plots showing the frequency of Tim-4^−^CD4^−^-, Tim-4^−^CD4^+^-, and Tim-4^+^CD4^+^-expressing cells within the CD163^−^ and CD163^+^ subsets of total small intestinal macrophages from WT mice. Right: Frequency of Tim-4^−^CD4^−^-, Tim-4^−^CD4^+^-, and Tim-4^+^CD4^+^-expressing cells within the CD163^−^ and CD163^+^ subsets of total small intestinal macrophages from WT mice. Data (*n* = 4 per group) are pooled from two independent experiments. Statistical comparisons were performed with an unpaired *t* test with Welch’s correction for parametric data and a Mann–Whitney test for nonparametric data. *, P ≤ 0.05; **, P ≤ 0.01. **(B)** Principal component analysis (PCA) of global gene expression from CD163^−^ and CD163^+^ subsets of Tim-4^−^CD4^−^, Tim-4^−^CD4^+^, and Tim-4^+^CD4^+^ macrophage of the small intestine isolated by FACS from five pooled WT mice, from at least three independent sorts. **(C)** Gene expression profile of the 3,206 genes differentially expressed (P-adjusted <0.001) in CD163^−^ and CD163^+^ subsets of Tim-4^−^CD4^−^, Tim-4^−^CD4^+^, and Tim-4^+^CD4^+^ macrophage of the small intestine with clusters identified by k-means clustering. **(D)** Top GO terms associated with each of the four clusters formed by the 3,206 DEGs. **(E)** Heatmaps showing expression profiles of genes in the top two pathways identified by PANTHER pathway analysis for clusters 1 and 2.

Previously, we and others have suggested that CD4 can be used as a maturation marker for gut macrophages (Schridde et al., 2017; Shaw et al., 2018). In agreement with this, *Cd4* was largely absent in cluster 0, the cluster dominated by recently arrived WT *Ccr2*^+^ macrophages, yet present in most other clusters (Fig. S2 A). We, therefore, investigated whether this maturation marker could be used across both the CD163^+^ compartment and CD163^−^ compartment to understand ontogeny and maturation. Indeed, assessment of Tim-4, CD163, and CD4 expression allowed identification of six subsets (Fig. 2 A), putatively reflecting longevity, location, and maturation, respectively. Notably, CD4^−^ cells were at a higher frequency in the LP, suggesting there is a greater proportion of high-turnover cells in this location (Fig. 2 A). Reciprocally, Tim-4^+^ cells were most frequent within the CD163^+^ S/M compartment (Fig. 2 A).

To confirm that identifying cells in this manner could be broadly useful in identifying cells of different maturity and sub-tissular niche, we sorted each of these populations and undertook bulk RNA-seq. Principle component analysis revealed that CD163 expression, along with CD163 associated genes, was responsible for the largest separation (PC1) between these six macrophage subsets, suggesting that sub-tissular location was the biggest factor controlling differential gene expression of total intestinal macrophages (Fig. 2 B). The second largest factor (PC2) driving separation of these subsets was the maturity of the cells, as determined by the expression of CD4, though this was most evident in the CD163^−^ subsets (Fig. 2 B). One possible explanation for this, in line with our scRNA-seq pseudotime analysis, is that acquisition of an “LP” signature takes longer than an “S/M” signature. This analysis confirms that greater heterogeneity is seen within the LP macrophages (CD163^−^) than the S/M macrophages (CD163^+^) (De Schepper et al., 2018).

Unsupervised hierarchical k-means clustering of 3,206 DEGs (P-adjusted <0.001) generated four clusters (Fig. 2 C and Table S1). Genes upregulated in all CD163^+^ populations were found in cluster 1 and include *Bmp2*, *F13a1*, *Mrc1*, *Lyve1*, *Cd36*, and *Folr2*, supporting their identity as S/M macrophages (Chakarov et al., 2019; Gabanyi et al., 2016; Kang et al., 2020; Muller et al., 2014). Expression of genes in this cluster was similar across all three populations identified by expression of CD4 and Tim-4. This demonstrated that although CD163^+^ macrophages can be split into three populations based on CD4 and Tim-4, all these populations have a strong muscularis signature (Fig. 2 C and Table S1).

Similarly, cluster 2 defined an LP signature evident in all CD163^−^ populations and included *Itgax* (CD11c), a marker previously used to distinguish between LP and muscularis macrophages (Muller et al., 2014). Within this cluster, Tim-4^−^CD4^−^ cells had lower expression of cluster 2–associated LP signature genes than the Tim-4^–^CD4^+^ and Tim-4^+^CD4^+^ cells, suggesting that they were less differentiated than the CD4– and Tim-4–expressing subsets (Fig. 2 C). This concept was reinforced by cluster 3, which consisted of genes which are reported to be highly expressed in mature or long-lived small intestinal macrophages, such as *Cd4*, *Dtx3*, *Hes1*, *Timd4*, and *Cd63* (De Schepper et al., 2018; Gross-Vered et al., 2020; Schridde et al., 2017; Shaw et al., 2018). The CD4^−^ LP macrophages expressed much lower levels of these genes than CD4^+^ LP macrophages, and there was an expression gradient suggesting gradual upregulation of these genes as the cells mature (Fig. 2 C and Table S1).

Finally, cluster 4 contained genes most highly expressed in CD163^−^CD4^−^Tim4^−^ cells, associated with recent arrival into the intestine and early differentiation from monocyte to macrophage (*Ly6c2*, *Ccr2*, *Id3*, *Bach1*, and *Nfkb1*) (Dick et al., 2022; Gross-Vered et al., 2020; Schridde et al., 2017; Shaw et al., 2018), supporting the idea that the CD4^–^Tim-4^–^CD163^–^ cells are the most recently monocyte-derived cells (Fig. 2 C and Table S1). Furthermore, overlaying the gene signature from each sorted population onto our scRNA-seq dataset further confirmed that the CD163^−^CD4^−^Tim4^−^ population aligned most closely with the *Ccr2*-enriched clusters, 0 and 13 (Fig. S2 B). As expected, gene signatures for all three sorted CD163^+^ populations overlaid with the *Cd163*-enriched scRNA-seq clusters 2 and 9, i.e., those with the highest serosal signature (Fig. S2 B). The CD163^−^CD4^+^Tim4^−^ and CD163^−^CD4^+^Tim4^+^ populations closely aligned with clusters 1, 4, and 7, i.e., those with the highest LP signature (Fig. S2 B).

Top gene ontology (GO) terms for genes within the CD163^+^-associated cluster 1 were generally related to intracellular signalling (NIK/NF-κB signalling, negative regulation of MAPK cascade, and calcium-mediated signalling), and leukocyte migration (granulocyte chemotaxis) (Fig. 2 D). Top GO terms for cluster 2, associated with CD163^−^ macrophages, were related to protein transport (protein targeting to lysosome) and lipid metabolism (sterol biosynthetic process; cholesterol transport) T cell proliferation (negative regulation of T cell proliferation), TGF-β signalling (transforming growth factor β receptor signalling pathway), and integrin signalling (integrin-mediated signalling pathway) (Fig. 2 D). Only one GO term was associated with cluster 3, suggesting involvement in lipid metabolism. In line with recent monocyte entry into the tissue, cluster 4 was associated with GO terms relating to actin assembly and cell migration (Fig. 2 D). Protein ANalysis THrough Evolutionary Relationships (PANTHER) pathway analysis further suggested that CD163^+^ macrophages (cluster 1) are involved in neuron development (axon guidance mediated by semaphorins) and innate immune cell recognition of pathogen-associated molecular patterns (Toll receptor signalling pathway), and CD163^−^ (cluster 2) macrophages are involved in lipid synthesis (cholesterol biosynthesis and androgen/estrogen/progesterone biosynthesis) (Fig. 2 E). No pathways were identified from the genes in cluster 3, and pathway analysis of genes in cluster 4 supported the identification of CD163^−^Tim4^−^CD4^−^ cells as migrating cells (cytoskeleton regulation by Rho GTPase) with inflammatory potential (inflammation mediated by chemokine and signalling pathway) (Fig. S2 C).

Thus, a three-feature analysis of gut-resident macrophages (Tim-4, CD4, and CD163) identifies six unique populations defined by differences in location, maturity and, likely, longevity. However, there are differences in the types of populations these markers will define between the LP and muscularis. In the LP, three distinct populations will be defined: (1) CD4^−^Tim-4^−^ that appears to be recently monocyte-derived and poorly differentiated; (2) CD4^+^Tim-4^−^ macrophages that have a strong LP signature but weaker expression of longevity markers than Tim-4^+^ macrophages; and (3) CD4^+^Tim-4^+^ macrophages that express the highest levels of longevity markers. Contrasting this, although CD4^−^Tim-4^−^, CD4^+^Tim-4^−^, and CD4^+^Tim-4^+^ populations are evident by flow cytometry within the CD163^+^ muscularis macrophage population, these subsets have highly similar transcriptional profiles, and all express strong muscularis gene signatures.

Previously, we demonstrated that long-lived RTMs could be identified by expression of Tim-4 (Shaw et al., 2018). However, we had not distinguished between macrophages in the LP or S/M. Therefore, to formally assess the longevity of our newly identified macrophage populations, we dosed adult *Cx3cr1*^creER^*R26-yfp* mice with tamoxifen for five consecutive days, as previously described (Shaw et al., 2018; Yona et al., 2013). At 5 days, 16, 24, and 32 wk after the final tamoxifen dose, mice were sacrificed to assess the expression of YFP within CD163^+^ and CD163^−^ macrophage subsets.

**Figure 3.**
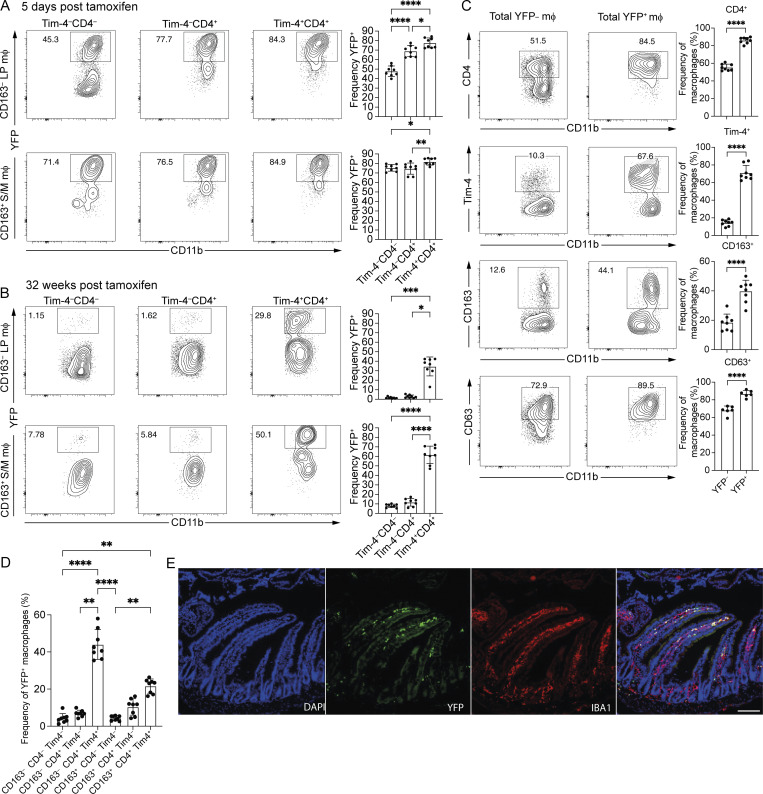
**Long-lived macrophages are present in the LP and S/M. (A)** Left: Representative flow cytometry plots showing the frequency of YFP-expressing cells within the CD163^−^ and CD163^+^ subsets of Tim-4^−^CD4^−^, Tim-4^−^CD4^+^, and Tim-4^+^CD4^+^ macrophage of the small intestine of *Cx3cr1*^creER^*:R26-yfp* mice, 5 days after tamoxifen treatment. Right: Frequency of YFP-expressing cells within the CD163^–^ and CD163^+^ subsets of Tim-4^–^CD4^−^, Tim-4^−^CD4^+^, and Tim-4^+^CD4^+^ macrophage of the small intestine of *Cx3cr1*^creER^*:R26-yfp* mice, 5 days after tamoxifen treatment. **(B)** Left: Representative flow cytometry plots showing the frequency of YFP-expressing cells within the CD163^−^ and CD163^+^ subsets of Tim-4^−^CD4^−^, Tim-4^–^CD4^+^, and Tim-4^+^CD4^+^ macrophage of the small intestine of *Cx3cr1*^creER^*:R26-yfp* mice, 32 wk after tamoxifen treatment. Right: Frequency of YFP-expressing cells within the CD163^−^ and CD163^+^ subsets of Tim-4^−^CD4^−^, Tim-4^–^CD4^+^, and Tim-4^+^CD4^+^ macrophage of the small intestine of *Cx3cr1*^creER^*:R26-yfp* mice, 32 wk after tamoxifen treatment. **(C)** Left: Representative flow cytometry plots showing expression of CD4, Tim-4, CD163, or CD63 in YFP^+^ and YFP^−^ macrophages of the small intestine from *Cx3cr1*^creER^*:R26-yfp*, 32 wk after tamoxifen treatment. Right: Frequency of YFP^+^ and YFP^–^ macrophages expressing CD4, Tim-4, CD163, or CD63 in the small intestine of *Cx3cr1*^creER^*:R26-yfp* mice, 32 wk after tamoxifen treatment. **(D)** Frequency of total YFP^+^ macrophages expressing CD163, CD4, and Tim-4 in the small intestine of *Cx3cr1*^creER^*:R26-yfp* mice, 32 wk after tamoxifen treatment. **(E)** Left: Representative immunofluorescence of small intestine section from *Cx3cr1*^creER^*:R26-yfp*, 32 wk after tamoxifen treatment. DAPI (blue), IBA1 (red), and YFP (green). Scale bar = 100 μm. **(A–C)** Numbers in flow cytometry plots denote the percentages of cells within the gate. **(A–D)** Data (*n* = 6–8 per group) are pooled from two to three independent experiments. Error bars show mean ± SD. Statistical comparisons between two groups were performed with an unpaired *t* test with Welch’s correction for parametric data and a Mann–Whitney test for nonparametric data. Statistical comparisons between more than two groups were performed with a one-way ANOVA, with Tukey’s multiple comparison test for parametric data and a Kruskal–Wallis test with Dunn’s multiple comparison test for nonparametric data. *, P ≤ 0.05; **, P ≤ 0.01; ***, P ≤ 0.001; and ****, P ≤ 0.0001.

**Figure S3.**
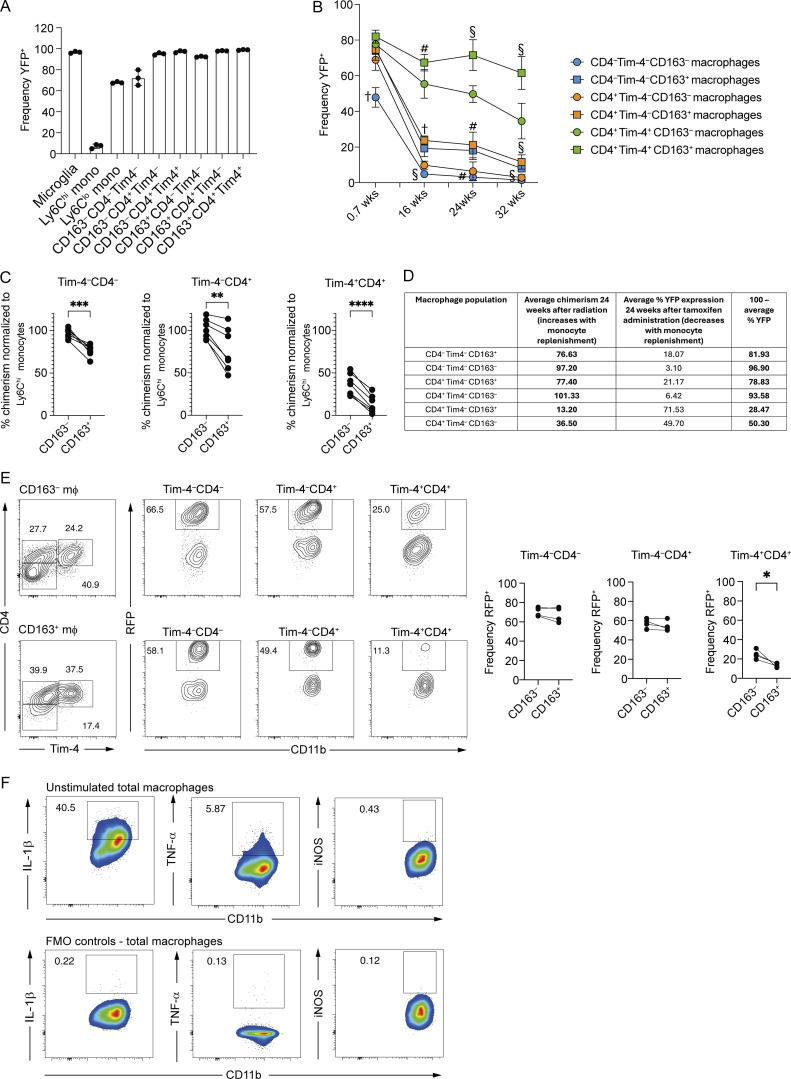
**Determination of monocyte contribution to small intestinal macrophage subsets in fate-mapping reporter mice and gut-shielded chimeras and biological and technical controls for *ex vivo* macrophage stimulations. (A)** Frequency of YFP-expressing microglia, blood monocytes, and small intestinal macrophages 1 day after tamoxifen treatment of *Cx3cr1*^creER^*:R26-yfp* mice. Data from *n* = 3 per group. **(B)** Frequency of YFP-expressing small intestinal macrophages at 0.7 (5 days), 16, 24, and 32 wk after tamoxifen treatment of *Cx3cr1*^creER^*:R26-yfp* mice. Data (*n* = 6–8 per group) are pooled from two (16 and 24 wk) or three (0.7 and 32 wk) independent experiments. **(C)** Frequency of donor-derived cells in small intestinal macrophages from abdomen shielded chimeras, 24 wk after irradiation and reconstitution with congenic bone marrow cells, normalized to the chimerism of Ly6C^hi^ blood monocytes. Data (*n* = 7 per group) are pooled from three harvests, from two independently generated chimera cohorts. **(D)** Table comparing monocyte replenishment of macrophages using gut-shielded chimeras and tamoxifen-induced fate mapping reporter mice. **(E)** Left: Representative flow cytometry plots showing the frequency of RFP-expressing cells within the CD163^−^ and CD163^+^ subsets of Tim-4^−^CD4^−^, Tim-4^−^CD4^+^, and Tim-4^+^CD4^+^ macrophage of the small intestine of 5-mo-old *Ms4a3*^Cre^*-R26*^*TdT*^ mice. Right: Frequency of RFP-expressing cells within the CD163^−^ and CD163^+^ subsets of Tim-4^–^CD4^−^, Tim-4^–^CD4^+^, and Tim-4^+^CD4^+^ macrophage of the small intestine of *Ms4a3*^Cre^*-R26*^*TdT*^ mice. Data are pooled from four mice. **(F)** Top: Representative flow cytometry plots showing the frequency of unstimulated macrophages expressing pro-IL-1β, TNF-α, or iNOS from small intestinal cells enriched for macrophages. Bottom: Representative flow cytometry plots showing fluorescence minus one (FMO) controls for stimulated macrophages from small intestinal cells enriched for macrophages. **(A and B)** Error bars show mean ± SD. **(B)** Statistical comparisons were performed with an unpaired *t* test with Welch’s correction for parametric data and a Mann–Whitney test for nonparametric data. Significance is shown for comparisons between CD163^+^ and CD163^−^ counterparts within the Tim-4^−^CD4^−^, Tim-4^−^CD4^+^, and Tim-4^+^CD4^+^ macrophage subsets at each time point. #, P ≤ 0.01, §, P ≤ 0.001, †, P ≤ 0.0001. **(C)** Statistical comparisons were performed with a paired *t* test. *, P ≤ 0.05; **, P ≤ 0.01; ***, P ≤ 0.001; ****, P ≤ 0.0001.

At 5 days after cessation of tamoxifen treatment, YFP expression within CD163^+^ macrophages (Tim-4^−^CD4^−^, Tim-4^−^CD4^+^, and Tim-4^+^CD4^+^) was uniformly >70% on average and similar to that seen in two of the CD163^–^ populations (Tim-4^–^CD4^+^ and Tim-4^+^CD4^+^) (Fig. 3 A). Labelling of CD163^–^Tim-4^−^CD4^−^ macrophages was, however, lower than seen in all other subsets, likely due to their more recent differentiation from monocytes, associated with more recent upregulation of CX3CR1 (Yona et al., 2013). In agreement with CD163^−^Tim-4^−^CD4^−^ macrophages being the most recently differentiated from monocytes, assessment of YFP expression by blood monocytes and intestinal macrophages on day 1 after cessation of 5 consecutive days of tamoxifen treatment showed that YFP was detectable in <5% of Ly6C^hi^ blood monocytes and in ∼70% of Ly6C^lo^ blood monocytes and CD163^−^Tim-4^−^CD4^−^ macrophages (Fig. S3 A). By 32 wk, the majority of Tim-4^−^ macrophages, both CD163^+^ and CD163^−^, had been replaced by YFP^–^ cells (Fig. 3 B). Tim-4^+^ macrophages still comprised a significant proportion of YFP^+^ cells, regardless of CD163 expression status, and are, therefore, likely present in the LP and S/M (Fig. 3 B). These data confirm the utility of Tim-4 as a marker of self-maintaining macrophages in different compartments of the small intestine and at longer time points than we previously showed (Shaw et al., 2018).

At 16, 24, and 32 wk, CD163 expression further differentiated the Tim-4^+^ macrophages, with CD163^+^Tim4^+^ macrophages retaining a higher frequency of YFP^+^ cells than their CD163^−^ counterparts (Fig. 3 B and Fig. S3 B). CD163 expression also differentiated the Tim-4^−^ macrophage subsets at these time points, though YFP expression was not more than 24% on average in any of these populations (Fig. S3 B). These findings were further confirmed by the generation of shielded bone marrow chimeras, as previously described (Jenkins et al., 2011; Shaw et al., 2018) (Fig. S3, C and D), and through the use of 5-mo-old *Ms4a3*^Cre^*-R26*^*TdT*^ mice (Liu et al., 2019) (Fig. S3 E). Together, these complementary approaches confirm that monocyte-derived macrophages are slowly incorporated into the Tim-4^+^ RTM population over time (Liu et al., 2019) and further refine our understanding of the contribution of monocyte-derived cells to the small intestinal RTM pool by demonstrating that monocyte-derived cells contribute more to the CD163^–^Tim-4^+^ than the CD163^+^Tim-4^+^ population.

When gating on total YFP^+^ macrophages at 32 wk, we observed that they were characterized by higher expression of CD4, Tim-4, and CD163 than total YFP^–^ macrophages (Fig. 3 C), suggesting that they are useful markers of longevity. CD63, a marker recently identified as a potential marker of long-lived, self-renewing intestinal RTMs (De Schepper et al., 2018), was most highly expressed by YFP^+^ macrophages but was also expressed by a majority of YFP^–^ macrophages (Fig. 3 C), potentially limiting its utility as a specific marker of long-lived intestinal RTMs.

Furthermore, when total YFP^+^ macrophages at 32 wk were assessed for expression of CD163, CD4, and Tim-4, the greatest proportion of YFP^+^ macrophages were found to be CD163^−^CD4^+^Tim-4^+^ (44.0 ± 8.0%), followed by CD163^+^CD4^+^Tim-4^+^ (21.6 ± 3.8%) (Fig. 3 D). This led us to predict that a striking proportion of long-lived macrophages would be located within the LP, as well as the S/M, and not just the S/M as previously suggested (De Schepper et al., 2018). We, therefore, examined the location of YFP^+^ macrophages within the small intestine of mice 32 wk after tamoxifen treatment and found that these macrophages were indeed located in both sub-tissular compartments (Fig. 3 E).

These data demonstrate that in both the LP (CD163^−^) and the S/M (CD163^+^), Tim-4 identifies the macrophages with the lowest dependency on monocytic replenishment. Although the longest-lived macrophages were those Tim-4^+^ cells present in the S/M, even at 32 wk there was a population of YFP^+^ macrophages in the LP that had not turned over from monocytes, demonstrating that there are long-lived macrophages in both the LP and S/M regions of the tissue.

Much of our understanding of the function of intestinal RTMs has been based on whole mixed preparations of digested LP and muscularis (Hadis et al., 2011; Medina-Contreras et al., 2011), prior to our understanding of long-lived intestinal macrophages (De Schepper et al., 2018; Shaw et al., 2018). Alternatively, muscularis macrophages have been isolated utilizing a gut-splitting approach (Gabanyi et al., 2016; Matheis et al., 2020). Making use of our new marker of location (CD163) and longevity marker (Tim-4), we sought to establish whether there were differences between the functions of the longest-lived macrophage populations in the LP and the muscularis in a mixed culture. To this end, single-cell suspensions from the intestine were cultured with vehicle control (Fig. S3 F) or proinflammatory stimuli and assessed for expression of pro-IL-1β, TNF-α, and inducible nitric oxide synthase (iNOS).

**Figure 4.**
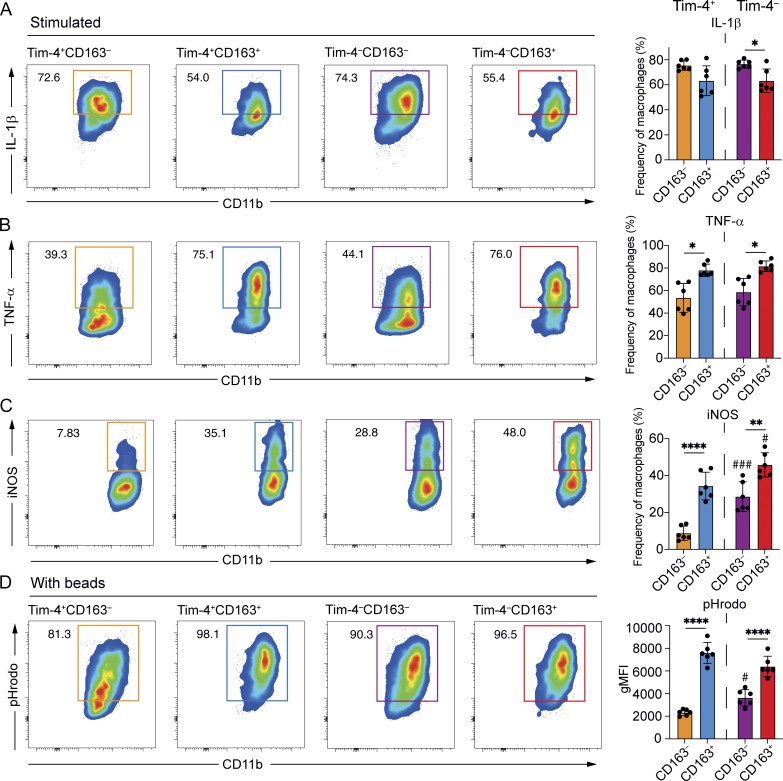
**Sub-tissular location is a dominant determinant of small intestinal macrophage function. (A)** Left: Representative flow cytometry plots showing the frequency of cells expressing pro-IL-1β from small intestinal cells enriched for macrophages and stimulated for 3 h. Right: Frequencies of stimulated macrophages expressing pro-IL1 β. **(B)** Left: Representative flow cytometry plots showing the frequency of cells expressing TNF-α from small intestinal cells enriched for macrophages and stimulated for 3 h. Right: Frequencies of stimulated macrophages expressing TNF-α. **(C)** Left: Representative flow cytometry plots showing the frequency of cells expressing iNOS from small intestinal cells enriched for macrophages and stimulated overnight. Right: Frequencies of stimulated macrophages expressing iNOS. **(D)** Left: Representative flow cytometry plots showing the frequency of pHrodo^+^ cells after 40-min incubation with pHrodo beads. Right: Geometric mean fluorescence intensity (gMFI) of pHrodo expression in macrophages incubated with beads. **(A–D)** Numbers in flow cytometry plots denote the percentages of cells within the gate. Error bars show mean ± SD. Data (*n* = 6 per group) are pooled from at least two independent experiments. Statistical comparisons were performed with a with one-way ANOVA, with Tukey’s multiple comparison test for parametric data and a Kruskal–Wallis test with Dunn’s multiple comparison test for nonparametric data. Significance is shown for comparisons between Tim-4^+^ and Tim-4^−^ macrophages within the same compartments: *, P ≤ 0.05; **, P ≤ 0.01; and ****, P ≤ 0.0001; and between Tim-4^+^ and Tim-4^−^ macrophages across compartments: #, P ≤ 0.01; ###, P ≤ 0.001.

We initially focussed on whether location would impact responsiveness to proinflammatory stimuli. For long-lived (Tim-4^+^) macrophages, location did not significantly impact production of pro-IL-1β in the presence of LPS (Fig. 4 A). Short-lived (Tim-4^−^) LP (CD163^−^) macrophages, however, were more likely to produce pro-IL-1β than their S/M (CD163^+^) counterparts (Fig. 4 A). Contrasting this, S/M macrophages expressed higher levels of both TNF-α and iNOS than LP macrophages for both long- and short-lived populations (Fig. 4, B and C). Thus, the sub-tissular location from which intestinal RTMs come has an impact on their propensity to produce proinflammatory factors, when equally exposed to stimuli in a mixed culture system.

As well as effects of sub-tissular location, we were also able to investigate functionality of shorter-lived macrophage populations (Tim-4^−^) compared with long-lived populations (Tim-4^+^) in the same cultures. Production of TNF-α and pro-IL-1β was independent of longevity (Fig. 4, A and B). However, iNOS production was enhanced in Tim-4^−^ populations from both the LP (CD163^–^) and muscularis (CD163^+^), suggesting that longevity or time spent in the tissue, as well as sub-tissular location, is an important determinant of iNOS production (Fig. 4 C). For iNOS, this resulted in long-lived LP (Tim-4^+^CD163^−^) macrophages producing strikingly low levels of iNOS compared with the other three macrophage subsets (Fig. 4 C).

Phagocytic capacity is another important functional feature of intestinal macrophages (Bain et al., 2014; Bujko et al., 2018). We, therefore, directly assessed phagocytosis *ex vivo* by culturing single-cell suspensions with pHrodo BioParticles. As for proinflammatory cytokine production, detectable differences in phagocytic capacity were evident between Tim-4^+^ LP (CD163^−^) and S/M (CD163^+^) macrophages, with LP macrophages displaying a reduced phagocytic capacity (Fig. 4 D). In comparison with location, longevity had a smaller effect on phagocytic capacity, and only within the LP compartment, highlighting location as the major factor determining differences in phagocytic functionality (Fig. 4 D).

Thus, when activated under the same conditions, detectable differences in macrophage function can be observed based on both location and longevity; iNOS in particular is impacted by both location and longevity in the LP.

**Figure 5.**
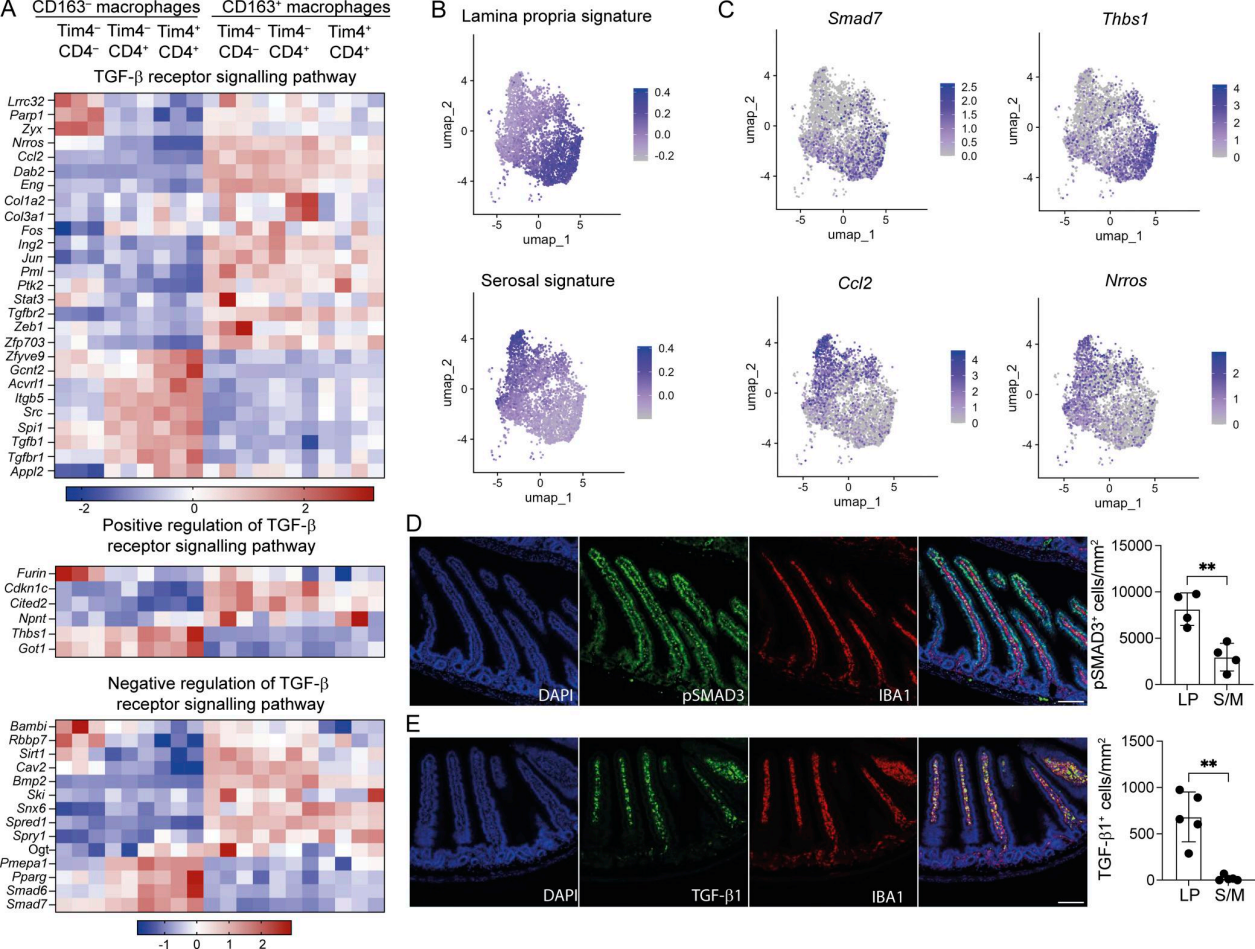
**TGF-β signalling pathways differ across sub-tissular locations of the small intestine. (A)** Heatmaps showing expression profiles of genes listed under the TGF-β receptor signalling pathway GO term (GO-0007179) and identified as a DEG by bulk RNA-seq. **(B)** UMAP plots showing cells expressing LP (top) or serosal (bottom) genes from ImmGen datasets. **(C)** UMAP plots showing (top) expression level of *Smad7* and *Thbs1* and (bottom) *Nrros* and *Ccl2*. **(D)** Left: Representative immunofluorescence image of small intestine section from WT mice. DAPI (blue), IBA1 (red), and pSMAD3 (green). Right: Quantification of pSMAD3-expressing cells in the LP and S/M of the small intestine in WT mice. **(E)** Left: Representative immunofluorescence image of small intestine section from WT mice. DAPI (blue), IBA1 (red), and TGF-β1 (green). Right: Quantification of TGF-β1–expressing cells in the LP and S/M of the small intestine in WT mice. **(D and E)** Data (*n* = 4–5 per group) are pooled from three independent experiments. Scale bar = 100 μm. Error bars show mean ± SD. Statistical comparisons were performed with an unpaired *t* test with Welch’s correction for parametric data and a Mann–Whitney test for nonparametric data. **, P ≤ 0.01. UMAP, uniform manifold approximation and projection.

In the intestine, a number of signals have been identified that are crucial to instruction of intestinal macrophage differentiation, including TGF-β, IL-10, lipid mediators, and short-chain fatty acids (Schridde et al., 2017; Scott et al., 2018; Zigmond et al., 2014). However, the impact of the same intestinal signals on resident macrophages in different intestinal tissue layers has not been explored, in part because it was not appreciated that long-lived macrophages are present in both the LP and S/M. Our bulk RNA-seq data allowed us to identify TGF-β receptor signalling pathways as a GO term (GO-0007179) that was differentially enriched between LP and S/M (Fig. 2, C and D). We further investigated the genes that contribute to GO-0007179, identifying known negative and positive regulators of TGF-β signalling. Interestingly, although enriched in the CD163^+^ LP macrophages (Fig. 2, C and D), a range of genes involved in TGF-β receptor signalling were found to be differentially expressed in macrophages within the LP (CD163^−^) and S/M (CD163^+^), including those involved in negative (*Smad6*; *Smad7*) and positive (*Furin*; *Thbs1*) regulation of this signalling pathway (Fig. 5 A).

**Figure S4.**
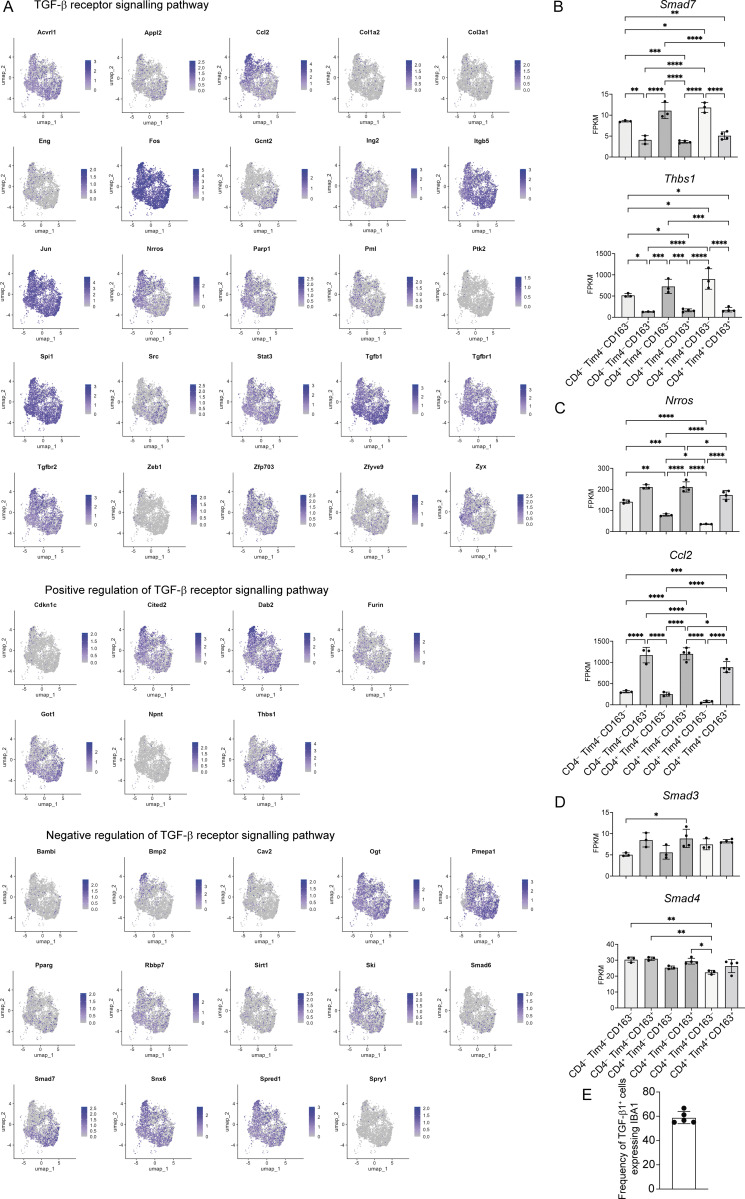
**Expression of genes from the TGF-β receptor signalling pathway GO term (GO-0007179) in small intestinal macrophage by scRNA-seq. (A)** UMAP plots showing expression of genes listed under the TGF-β receptor signalling pathway GO term (GO-0007179) and identified as a DEGs by bulk RNA-seq. **(B)** Expression of *Smad7* and *Thbs1* in six macrophage populations sorted from WT small intestinal tissues by bulk RNA-seq. **(C)** Expression of *Ccl2* and *Nrros* in six macrophage populations sorted from WT small intestinal tissues by bulk RNA-seq. **(D)** Expression of *Smad3* and *Smad4* in six macrophage populations sorted from WT small intestinal tissues by bulk RNA-seq. **(E)** Frequency of TGF-β1^+^ cells that co-express IBA1 in the small intestine of WT mice. Data (*n* = 5) are pooled from three independent experiments. Error bars show mean ± SD. Statistical comparisons between more than two groups were performed with a one-way ANOVA, with Tukey’s multiple comparison test for parametric data and a Kruskal–Wallis test with Dunn’s multiple comparison test for nonparametric data. *, P ≤ 0.05; **, P ≤ 0.01; ***, P ≤ 0.001; and ****, P ≤ 0.0001. UMAP, uniform manifold approximation and projection.

Furthermore, we investigated expression of all genes contributing to GO-0007179 across our scRNA-seq small intestinal macrophage dataset. Some genes identified by bulk RNA-seq were not detected by scRNA-seq due to low detection levels. Many genes were expressed throughout the macrophage pool, though some were restricted to clusters that scored more highly for LP or serosal macrophage signatures (Fig. 5, B and C; and Fig. S4 A).

Interestingly, *Smad7*, which was initially reported as a gene expressed early in response to TGF-β (Nakao et al., 1997), and *Thbs1*, reported to be an activator of TGF-β signalling (Crawford et al., 1998), were selectively upregulated in the macrophages scoring highly for the LP signature (Fig. 5, B and C; and Fig. S4 B). In contrast, *Ccl2,* reported to be downstream of TGF-β signalling in breast cancer (Gorbacheva et al., 2021), and *Nrros,* a myeloid-specific activator of TGFβ1-signalling (Qin et al., 2018), were more highly expressed in the macrophages with a high serosal signature (Fig. 5 B and C; and Fig. S4 C). Other genes, downstream of TGF-β signalling, such as *Smad3* and *Smad4*, were more similarly expressed across sub-tissular locations and subsets (Fig. S4 D), likely reflecting the importance of their phosphorylation and activation status in TGF-β signal transduction rather than their expression levels (Huang and Chen, 2012). These data suggest the possibility that the availability and/or activation of latent-TGF-β into its active form may differ across macrophages in the LP and S/M. Detection of phosphorylated SMAD3 (pSMAD3) in the LP and S/M compartments suggests that TGF-β signalling is active in both compartments (Fig. 5 D). However, in line with other recent reports (Chen et al., 2023; Reina-Campos et al., 2025), we observed a gradient of TGF-β1 expression, with high expression in the LP and little expression in the S/M (Fig. 5 E). Furthermore, the majority of the TGF-β1 expression was localized to ionized calcium-binding adapter molecule 1 (IBA1)^+^ macrophages (Fig. S4 E). Detection of pSMAD3 in the S/M in the absence of TGF-β1 is likely the result of high levels of TGF-β-2/3 produced in this sub-tissular compartment (Reina-Campos et al., 2025; Viola et al., 2023).

Together, these results raise the intriguing possibility that RTMs in the LP and S/M have different exposure to, or ways of regulating, TGF-β signalling, promoting distinct TGF-β−dependent transcriptional responses within their respective sub-tissular locations. We, therefore, chose to further investigate TGF-β signalling as a pathway that could have important effects across the LP and S/M compartments.

**Figure 6.**
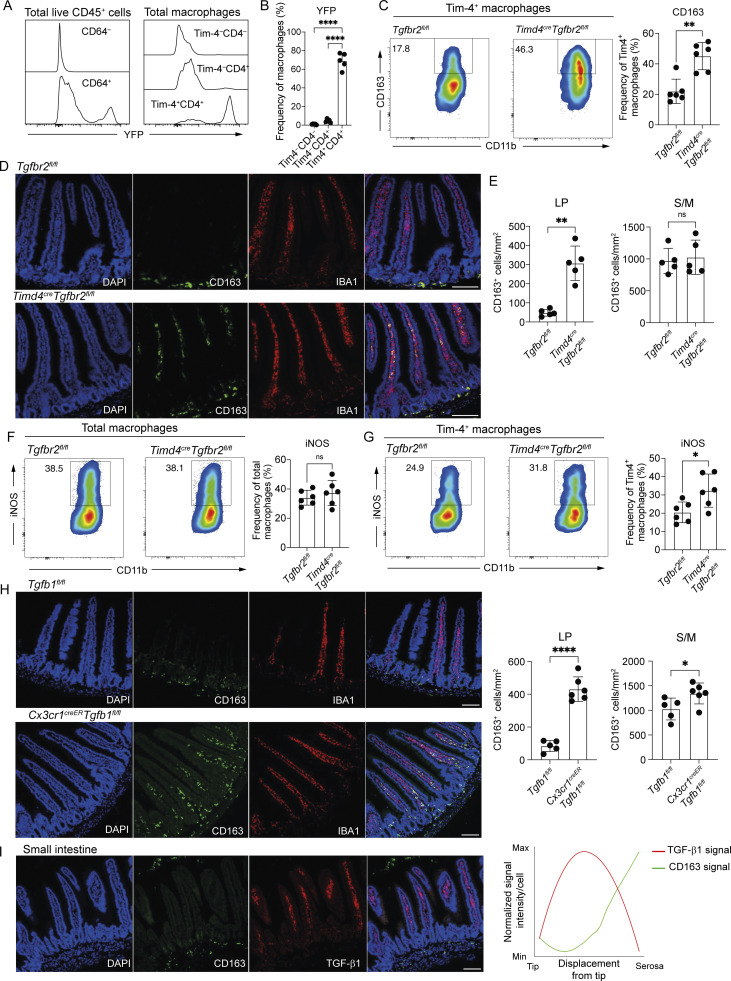
**TGF-β signalling controls the localization, phenotype, and responsiveness of long-lived small intestinal macrophages. (A)** Left: Expression of YFP in CD64^−^ and CD64^+^ cells from total live CD45^+^ cells in the small intestine. Right: Expression of YFP in Tim-4^−^CD4^−^, Tim-4^−^CD4^+^, and Tim-4^+^CD4^+^ macrophages in the small intestine. **(B)** Frequency of small intestinal Tim-4^−^CD4^−^, Tim-4^−^CD4^+^, and Tim-4^+^CD4^+^ macrophages expressing YFP. **(C)** Left: Representative flow cytometry plots showing the frequency of Tim-4^+^ macrophages expressing CD163 in *Timd4*^cre^*Tgfbr2*^fl/fl^ and *Tgfbr2*^fl*/*fl^ mice. Right: Frequencies of Tim-4^+^ macrophages expressing CD163 in *Timd4*^cre^*Tgfbr2*^fl/fl^ and *Tgfbr2*^fl*/*fl^ mice. **(D)** Representative immunofluorescence images of the small intestine showing the location of CD163-expressing macrophages in *Timd4*^cre^*Tgfbr2*^fl/fl^ and *Tgfbr2*^fl*/*fl^ mice. DAPI (blue), IBA1 (red), and CD163 (green). **(E)** Quantification of CD163-expressing cells in the LP (left) and S/M (right) of the small intestine in *Timd4*^cre^*Tgfbr2*^fl/fl^ and *Tgfbr2*^fl*/*fl^ mice. **(F)** Left: Representative flow cytometry plots showing the frequency of total macrophages expressing iNOS in response to overnight LPS and IFN-γ stimulation. Right: Frequencies of total macrophages expressing iNOS in response to overnight LPS and IFN-γ stimulation. **(G)** Left: Representative flow cytometry plots showing the frequency of Tim-4^+^ macrophages expressing iNOS in response to overnight LPS and IFN-γ stimulation. Right: Frequencies of Tim-4^+^ macrophages expressing iNOS in response to overnight LPS and IFN-γ stimulation. **(H)** Left: Representative immunofluorescence images of the small intestine showing the location of CD163-expressing macrophages in *Cx3cr1*^creER^*Tgfb1*^fl*/*fl^ and *Tgfb1*^fl*/*fl^ mice. DAPI (blue), CD163 (green), and IBA1 (red). Right: Quantification of CD163-expressing cells in the LP and S/M of the small intestine in *Cx3cr1*^creER^*Tgfb1*^fl*/*fl^ and *Tgfb1*^fl*/*fl^ mice. **(I)** Left: Representative immunofluorescence images of the small intestine showing the location of CD163- and TGF-β1–expressing cells in WT mice. DAPI (blue), CD163 (green), and TGF-β1 (red). Right: Quantification of CD163 and TGF-β1 expression levels in individual cells relative to their displacement from the tip. **(D, H, and I)** Scale bar = 100 μm. **(C, F, and G)** Numbers in flow cytometry plots denote the percentages of cells within the gate. Data (*n* = 6 per group) are pooled from two independent experiments. **(B, C, and E)** Data (*n* = 5 per group) are pooled from two independent experiments. **(H)** Data (*n* = 5–6 per group) are pooled from two independent experiments. Error bars show mean ± SD. **(I)** Data (*n* = 3 per group) are pooled from rom two independent experiments. SM, submucosa; M, muscularis externa; S, serosa. Statistical comparisons were performed with an unpaired *t* test with Welch’s correction for parametric data and a Mann–Whitney test for nonparametric data. *, P ≤ 0.05; **, P ≤ 0.01; ****, P ≤ 0.0001.

**Figure S5.**
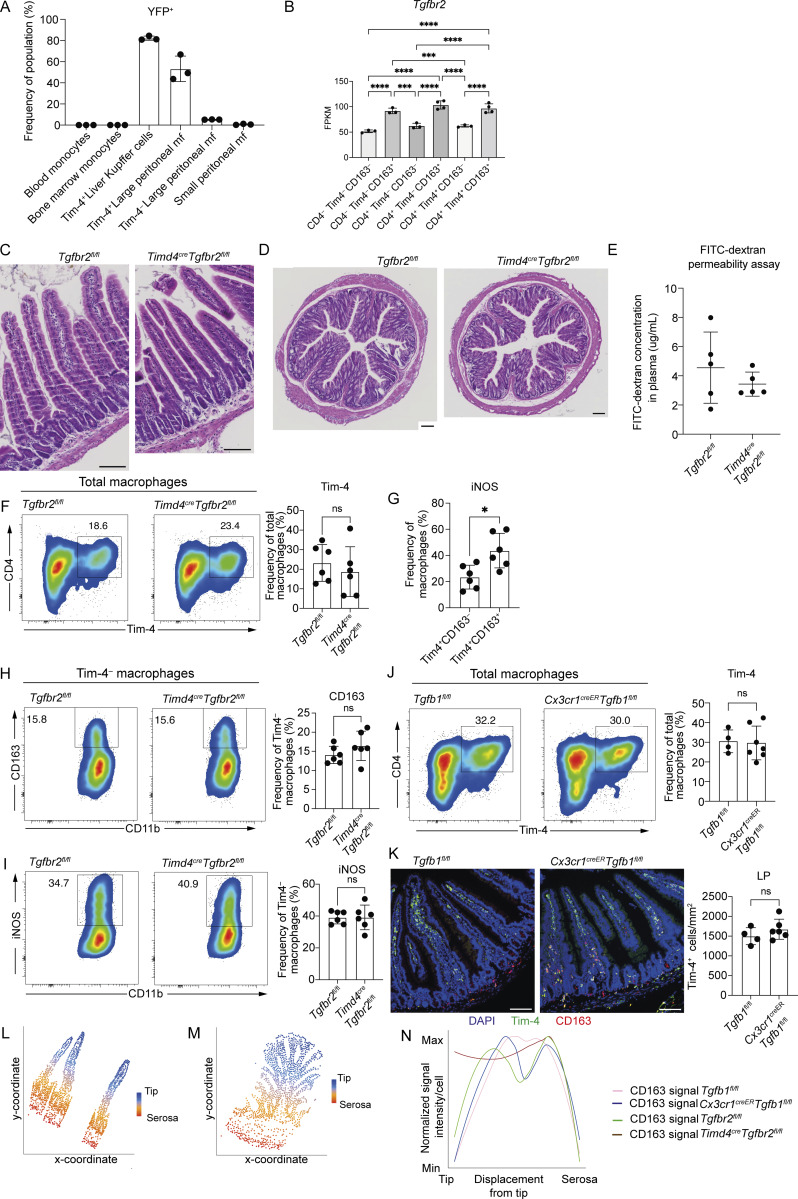
**Characterization of *Timd4*** ^**cre**^ ***Tgfbr2*** ^**fl/fl**^ **and *Cx3cr1*** ^**creER**^ ***Tgfb1*** ^**fl/fl**^ **mice. (A)** Frequency of Ly6C^hi^ blood and bone marrow monocytes, Tim-4^+^ liver Kupffer cells, Tim-4^+^ large peritoneal macrophages, Tim-4^−^ large peritoneal macrophages, and small peritoneal macrophages expressing YFP. Data (*n* = 3 per group) are from a single experiment. **(B)** Expression of *Tgfbr2* in six macrophage populations sorted from WT small intestinal tissues by bulk RNA-seq. **(C)** Representative H&E-stained small intestine from *Timd4*^cre^*Tgfbr2*^fl/fl^ mice and *Tgfbr2*^fl/fl^ mice. **(D)** Representative H&E-stained colon from *Timd4*^cre^*Tgfbr2*^fl/fl^ mice and *Tgfbr2*^fl/fl^ mice. **(E)** Concentration of FITC-dextran in plasma of mice 1 h after oral gavage with FITC-dextran. Data (*n* = 5 per group) are pooled from two independent experiments. **(F)** Left: Representative flow cytometry plots showing the frequency of small intestinal macrophages expressing Tim-4 in *Timd4*^cre^*Tgfbr2*^fl/fl^ and *Tgfbr2*^fl/fl^ mice. Right: Frequencies of Tim-4^+^ macrophages in the small intestine of *Timd4*^cre^*Tgfbr2*^fl/fl^ and *Tgfbr2*^fl*/*fl^ mice. **(G)** Frequency of Tim-4^+^CD163^−^ and Tim-4^+^CD163^+^ small intestinal macrophages expressing iNOS in response to overnight LPS and IFN-γ stimulation. **(H)** Left: Representative flow cytometry plots showing the frequency of Tim-4^−^ macrophages expressing CD163. Right: Frequencies of Tim-4^−^ macrophages expressing CD163. **(I)** Left: Representative flow cytometry plots showing the frequency of Tim-4^−^ macrophages expressing iNOS. Right: Frequencies of Tim-4^−^ macrophages expressing iNOS. **(J)** Left: Representative flow cytometry plots showing the frequency of Tim-4^+^ macrophages in the small intestine of *Cx3cr1*^creER^*Tgfb1*^fl/fl^ and *Tgfb1*^fl*/*fl^ mice. Right: Frequencies of Tim-4^+^ macrophages in the small intestine of *Cx3cr1*^creER^*Tgfb1*^fl/fl^ and *Tgfb1*^fl/fl^ mice. **(K)** Left: Representative immunofluorescence images showing the location of Tim-4– and CD163-expressing cells in the small intestine of *Cx3cr1*^creER^*Tgfb1*^fl/fl^ and *Tgfb1*^fl/fl^ mice. DAPI (blue), Tim-4 (green), and CD163 (red). Right: Quantification of Tim-4–expressing cells in the LP of the small intestine in *Cx3cr1*^creER^*Tgfb1*^fl/fl^ and *Tgfb1*^fl*/*fl^ mice. **(L)** Example of the spatial distribution plots used to measure the distance of individual cells from the serosa in the small intestine. **(M)** Example of the spatial distribution plots used to measure the distance of individual cells from the serosa in the colon. **(N)** Quantification of CD163 expression levels in individual cells relative to their displacement from the tip in the colon of adult *Cx3cr1*^creER^*Tgfb1*^fl/fl^ mice, 4 wk after tamoxifen dosing, adult *Timd4*^cre^*Tgfbr2*^fl/fl^, and respective Cre^–^ littermates. Data (*n* = 5–6 per group) are pooled from two independent experiments. **(C, D, and K)** Scale bar = 100 μm. **(F–K)** Data (*n* = 4–7 per group) are pooled from two independent experiments. Error bars show mean ± SD. Statistical comparisons between two groups were performed with an unpaired *t* test with Welch’s correction. *, P ≤ 0.05. Statistical comparisons between more than two groups were performed with a one-way ANOVA, with Tukey’s multiple comparison test. *, P ≤ 0.05; ***, P ≤ 0.001; and ****, P ≤ 0.0001.

Having identified long-lived Tim-4^+^ RTMs in the LP and S/M and demonstrated their differential transcriptional profile and responsiveness to proinflammatory stimuli, we wanted to determine whether their function was regulated by differential TGF-β signalling within these sub-tissular locations. Currently, no transgenic model exists to selectively target long-lived macrophages in the gut. We, therefore, generated a new *Timd4*^cre^ mouse. Although this mouse will impact long-lived macrophages at many sites (Dick et al., 2022), in the gut it will allow for selective targeting of the *Timd4*^+^ cells while sparing other populations. To confirm the specificity of the *Timd4*^cre^, we crossed this transgenic animal to the *R26-yfp* mouse (Srinivas et al., 2001), in which all cells that have expressed *Timd4*^cre^ will be labelled with YFP. As expected, YFP was highly expressed by intestinal CD64^+^ macrophages and not CD64^–^ cells and was well expressed by Tim-4^+^ macrophages (Fig. 6, A and B). Assessment of blood and bone marrow monocytes revealed no YFP expression, and high levels of YFP expression was seen in liver Tim-4^+^ Kupffer cells and Tim-4^+^ large peritoneal macrophages, but not Tim-4^−^ large peritoneal macrophages or small peritoneal macrophages (Fig. S5 A), thus confirming the specificity of our novel *Timd4*^cre^ mouse.

Given our interest in TGF-β signalling in the LP and S/M*, Timd4*^cre^ animals were crossed with *Tgfbr2*^fl/fl^ mice (Levéen et al., 2002) to delete TGF-β-receptor 2 (TGF-βR2) on long-lived Tim-4^+^ macrophages in both compartments. Although more highly expressed in CD163^+^ S/M subsets, *Tgfbr2* is also expressed by CD163^−^ LP subsets (Fig. S5 B), and both TGF-βR1 and TGF-βR2 are required to transduce TGF-β signalling through formation of a heterodimeric receptor (Huang and Chen, 2012), thus deletion of either receptor abrogates TGF-β signalling. Following the loss of TGF-βR2 on Tim-4^+^ macrophages, we observed no overt signs of spontaneous inflammation in the small intestine (Fig. S5 C) or colon (Fig. S5 D), no increase in intestinal permeability (Fig. S5 E), and no change in frequency of short- and long-lived cells, as determined by Tim-4 expression (Fig. S5 F). Within the Tim-4^+^ population, however, the frequency of CD163^+^ macrophages was increased in *Timd4*^cre^*Tgfbr2*^fl/fl^ mice compared with *Tgfbr2*^fl/fl^ controls (Fig. 6 C).

The increased frequency of Tim-4^+^ macrophages expressing CD163 in the small intestine of *Timd4*^cre^*Tgfbr2*^fl/fl^ animals suggested that in the absence of TGF-β-signalling, long-lived macrophages were preferentially located in the S/M. To begin to explore this, we imaged CD163-expressing macrophages in the small intestine of *Timd4*^cre^*Tgfbr2*^fl/fl^ compared with their *Tgfbr2*^fl/fl^ littermates (Fig. 6 D). Unexpectedly, the expression of CD163 cells was no longer restricted to the S/M but was also detected on macrophages in the LP (Fig. 6 E), suggesting that the increased frequency of Tim-4^+^ macrophages expressing CD163 was due to upregulation of CD163 on long-lived LP macrophages. Thus, in the small intestine, TGF-β-signalling in long-lived Tim-4^+^ macrophages is critical to ensure their normal phenotypic development and the restriction of CD163^+^ expression to S/M macrophages.

In Fig. 4 C, we showed that long-lived LP macrophages are less likely to produce iNOS than short-lived LP macrophages or S/M macrophages. As long-lived LP macrophages in *Timd4*^cre^*Tgfbr2*^fl/fl^ mice upregulate CD163 expression, usually associated with S/M macrophages, we asked whether long-lived LP macrophages also adopted functional similarities to S/M macrophages, such as increased iNOS production.

In global macrophages, there was no difference in iNOS production between *Timd4*^cre^*Tgfbr2*^fl/fl^ mice and *Tgfbr2*^fl/fl^ controls (Fig. 6 F). When we assessed only Tim-4^+^ long-lived macrophages, however, we observed an increase in iNOS production in *Timd4*^cre^*Tgfbr2*^fl/fl^ (Fig. 6 G), with the iNOS being produced mostly by the CD163^+^ macrophages (Fig. S5 G). As expected, as this was a *Timd4*^cre^ mouse, no increase in CD163 expression or iNOS induction was seen in Tim-4^−^ macrophages (Fig. S5, H and I).

Our results thus far focus on the response of intestinal RTMs to TGF-β signalling, but results in Fig. 5 E and Fig. S4, A and E suggest that intestinal RTMs are themselves a major source of TGF-β1. While TGF-β signalling is known to be a key determinant of intestinal macrophage differentiation (Schridde et al., 2017), and loss of TGF-β1 causes intestinal inflammation conserved across species (Kotlarz et al., 2018; Shull et al., 1992), the local source of TGF-β1 in the intestine is unclear with production variously reported from epithelial cells and myeloid cells (Atarashi et al., 2011; Kashiwagi et al., 2015). In the lung (Branchett et al., 2021) and brain (Bedolla et al., 2024), macrophage-derived TGF-β1 has been shown to regulate the development of homeostatic RTMs, so we hypothesized that this would be similar in the intestine. To investigate the dependency of intestinal RTMs on macrophage-derived TGF-β1, we dosed adult *Cx3cr1*^creER^*Tgfb1*^fl/fl^ mice with tamoxifen for five consecutive days to conditionally delete *Tgfb1* from adult intestinal RTMs and harvested the tissue 4 wk later. In line with a requirement for continual exposure to TGF-β1, deletion of *Tgfb1* from adult macrophages resulted in aberrant expression of CD163 on macrophages in the LP (Fig. 6 H), without altering the frequency of Tim-4^+^ macrophages (Fig. S5 J) or their numbers within the LP (Fig. S5 K). This result phenocopies the results from the *Timd4*^cre^*Tgfbr2*^fl/fl^ mice and supports the emerging paradigm that TGF-β1 signalling in RTMs occurs via an autocrine signalling mechanism (Bedolla et al., 2024), extending this mechanism to include intestinal macrophages.

A previous *in vitro* study in human monocytes suggested that TGF-β may be a negative regulator of CD163 expression by macrophages (Pioli et al., 2004). Our findings in the intestine suggest that even in complex tissue environments, where multiple signals could be influencing macrophage phenotype, CD163-expressing macrophages are largely excluded from areas of high TGF-β1 expression. This idea was further supported by assessment of the expression levels of TGF-β1 and CD163 for each cell in relation to its displacement from the villus- or crypt-tip in the small intestine (Fig. S5 K). Using this approach, we observed that expression of TGF-β1 was very low near the villus tip and then peaked in the villi before diminishing again at near the serosa, while CD163 showed an inverse pattern of expression (Fig. 6 I).

**Figure 7.**
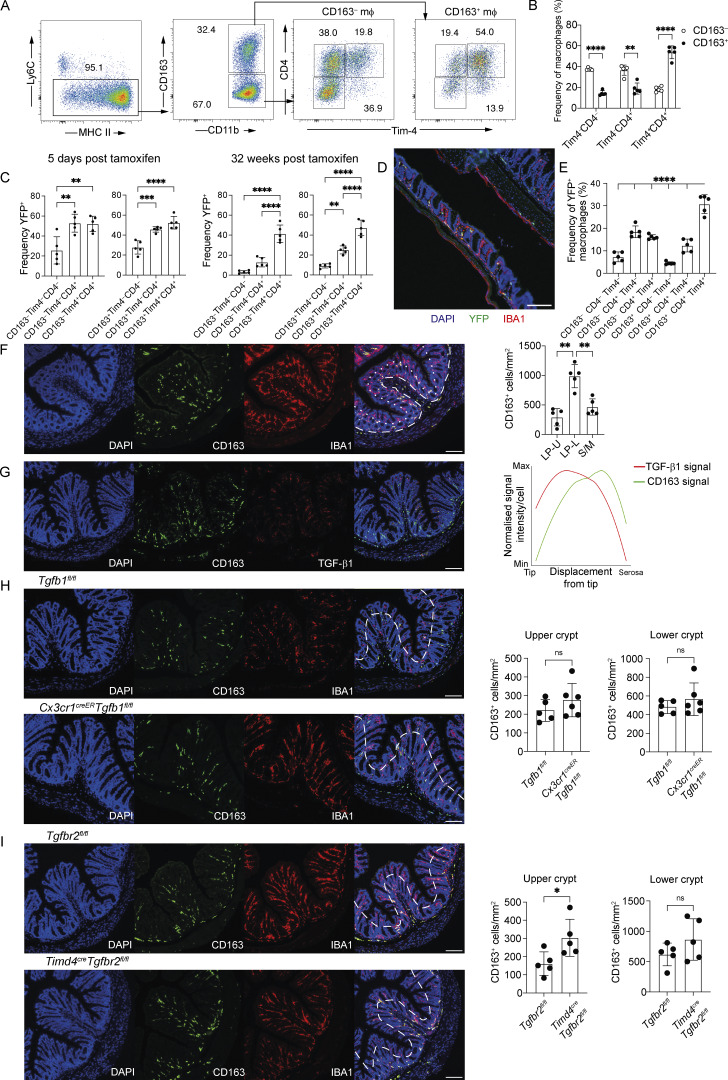
**Long-lived macrophages are present in the LP and S/M of the colon and are regulated by TGF-β signalling in the upper crypt region. (A)** Representative flow cytometry plots showing the frequency of Tim-4^−^CD4^−^-, Tim-4^−^CD4^+^-, and Tim-4^+^CD4^+^-expressing cells within the CD163^−^ and CD163^+^ subsets of total colonic intestinal macrophages from WT mice. **(B)** Frequency of Tim-4^−^CD4^−^-, Tim-4^−^CD4^+^-, and Tim-4^+^CD4^+^-expressing cells within the CD163^−^ and CD163^+^ subsets of colonic macrophages from WT mice. Numbers in flow cytometry plots denote the percentages of cells within the gate. **(C)** Frequency of YFP-expressing cells within the CD163^−^ and CD163^+^ subsets of Tim-4^−^CD4^–^, Tim-4^−^CD4^+^, and Tim-4^+^CD4^+^ macrophage of the colon from *Cx3cr1*^creER^*:R26-yfp* mice, 5 days and 32 wk after tamoxifen treatment. **(D)** Representative immunofluorescence of colon tissue from *Cx3cr1*^creER^*:R26-yfp* mice, 32 wk after tamoxifen treatment. DAPI (blue), IBA1 (red), and YFP (green). **(E)** Frequency of total YFP^+^ macrophages expressing CD163, CD4, and Tim-4 in the colon of *Cx3cr1*^creER^*:R26-yfp* mice, 32 wk after tamoxifen treatment. **(B, C, and E)** Data (*n* = 5 per group) are pooled from two independent experiments. For clarity, only comparisons to CD163^+^CD4^+^Tim4^+^ populations have been shown in E. **(F)** Left: Representative immunofluorescence image of colon section from WT mice showing the location of CD163-expressing macrophages. DAPI (blue), IBA1 (red), and CD163 (green). Right: Quantification of CD163^+^ macrophages in the S/M, and upper (LP-U) and lower (LP-L) layers of the LP. Dashed line demarcates the boundary between the S/M, and LP. Dotted line demarcates the boundary between the upper (LP-U) and lower (LP-L) layers of the LP. **(G)** Left: Representative immunofluorescence images of the colon showing the location of CD163- and TGF-β1-expressing cells in WT mice. DAPI (blue), CD163 (green), and TGF-β1 (red). Right: Quantification of CD163 and TGF-β1 expression levels in individual cells relative to their displacement from the crypt tip. Data (*n* = 3 per group) are pooled from two independent experiments. **(H)** Representative immunofluorescence images of the colon showing the location of CD163-expressing macrophages in *Cx3cr1*^creER^*Tgfb1*^fl/fl^ and *Tgfb1*^fl*/*fl^ mice. DAPI (blue), CD163 (green), and IBA1 (red). Right: Quantification of CD163-expressing cells in the LP and S/M of the colon in *Cx3cr1*^creER^*Tgfb1*^fl*/*fl^ and *Tgfb1*^fl*/*fl^ mice. **(I)** Representative immunofluorescence images of the colon showing the location of CD163-expressing macrophages in *Timd4*^cre^*Tgfbr2*^fl*/*fl^ and *Tgfbr2*^fl*/*fl^ mice. DAPI (blue), CD163 (green), and IBA1 (red). Right: Quantification of CD163-expressing cells in the LP and S/M of the colon in *Timd4*^cre^*Tgfbr2*^fl/fl^ and *Tgfbr2*^fl*/*fl^ mice. **(H and I)** Data (*n* = 5–6 per group) are pooled from two independent experiments. **(D and F–I)** Scale bar = 100 μm. Error bars show mean ± SD. Statistical comparisons between two groups were performed with an unpaired *t* test with Welch’s correction for parametric data and a Mann–Whitney test for nonparametric unpaired data. Statistical comparisons between more than two groups were performed with a one-way ANOVA, with Tukey’s multiple comparison test for parametric data and a Kruskal–Wallis test with Dunn’s multiple comparison test for nonparametric data. *, P ≤ 0.05; **, P ≤ 0.01; ***, P ≤ 0.001; and ****, P ≤ 0.0001.

The colon is physiologically and structurally different from the small intestine (Treuting et al., 2018). We, therefore, also undertook characterization of colonic macrophages to determine whether the six populations we had identified in the small intestine were similarly present and regulated by TGF-β1. Assessment of colonic macrophages for expression of CD163, Tim-4, and CD4 expression similarly allowed identification of six subsets (Fig. 7 A). As seen in the small intestine, the CD163^+^ macrophages contained a higher frequency of Tim-4^+^ macrophages than did the CD163^–^ macrophage population (Fig. 7 B). Comparing tamoxifen-induced expression of YFP in our six colonic macrophage populations at 5 days and 32 wk after the final tamoxifen dose in *Cx3cr1*^creER^*R26-yfp* mice, we observed similar results as those from the small intestine with Tim-4^+^ macrophages comprising a significant proportion of YFP^+^ cells, regardless of CD163 expression status (Fig. 7 C). In line with the small intestine, examination of the location of YFP^+^ macrophages in the colon of *Cx3cr1*^creER^*R26-yfp* mice 32 wk after tamoxifen treatment showed YFP^+^ macrophages located in both the LP and S/M (Fig. 7 D). We were, therefore, initially surprised to find that, in contrast to the small intestine, the greatest proportion of YFP^+^ macrophages were CD163^+^CD4^+^Tim-4^+^ rather than CD163^−^CD4^+^Tim-4^+^ (Fig. 7 E). The dominance of CD163^+^CD4^+^Tim-4^+^ over CD163^−^CD4^+^Tim-4^+^ within the YFP^+^ macrophage population in the colon could reflect the thicker muscularis layer of the colon, which we predicted to contain a higher number of CD163^+^ macrophages; however, in agreement with other reports (Hegarty et al., 2026; Kang et al., 2020), we found that CD163 expression is not restricted to the S/M in the colon but is also expressed by colonic LP macrophages, mostly in the lower crypt region, closer to the serosa (Fig. 7 F). Thus, the large population of macrophages expressing CD163 in the colonic LP accounts for the dominance of CD163^+^CD4^+^Tim-4^+^ macrophages within the YFP^+^ population of the colon.

Due to the observed differences in CD163 expression between the small intestine and colon, we investigated the relationship between TGF-β1 and CD163 expression in the colon. Unlike the small intestine, structural features of the colon include transverse folds in the mucosa, which create variation in the distance of cells located within the submucosa from the crypt tip or the serosa (Fig. 7 F). Nonetheless, we used our approach to quantify the expression of TGF-β1 and CD163 on each cell in relation to its displacement from the crypt tip (Fig. S5 L). Despite differences in location of CD163^+^ macrophages between the small intestine and colon, in agreement with our hypothesis that TGF-β1 negatively regulates CD163 expression in the intestine, TGF-β1 expression was graded within the LP of the colon, with higher expression in the upper crypt region, further from the serosa (Fig. 7 G). Observing an inverse gradient of TGF-β1 and CD163 expression in the colon, but a less clear physical boundary than in the small intestine, we probed whether deleting TGF-βR2 or TGF-β1, from long-lived and total intestinal adult RTMs, respectively, would have any effect on CD163 expression in the colon. In *Cx3cr1*^creER^*Tgfb1*^fl/fl^ mice, where *Tgfb1* was temporally deleted from all intestinal RTMs in adulthood, no change in CD163 expression was observed in the upper (upper-LP) or lower crypt (lower-LP + S/M) regions (Fig. 7 H and Fig. S5 M). However, in *Timd4*^cre^*Tgfbr2*^fl/fl^ mice, with constitutive deletion of *Tgfbr2,* only in long-lived RTMs, the number of CD163^+^ macrophages increased in the upper crypt, but not in the lower crypt region (Fig. 7 I and Fig. S5 M). Together, these data suggest that the contribution of TGF-β signalling to homeostatic macrophage development varies across intestinal tissue layers and compartments.

Thus, using our new phenotyping and transgenic approaches to explore the distinct tissular niches of gut macrophages, our data reveal a role for intestinal RTM-derived TGF-β1 in regulating small intestinal LP macrophage identity.

## Discussion

Until relatively recently, it was thought that all macrophages in the gut were constantly replenished from monocytes (Bain et al., 2014). In our previous publication (Shaw et al., 2018), we did not establish where Tim-4^+^ macrophages were located within the intestinal tissue, and based on subsequent publications, it was assumed that these cells were largely in the S/M (De Schepper et al., 2018). Indeed, a perspective has even been raised that monocyte replenishment rates in the LP are so high they should not be considered a niche (Viola and Boeckxstaens, 2021). Notably, publications by Kang et al. (Kang et al., 2020) and Chiaranunt et al. (2023) suggested that Tim-4^+^ macrophages may also be in multiple sub-tissular niches within the colon, but this was not fully explored with respect to LP versus S/M localization.

Making use of scRNA-seq from WT mice and *Ccr2*^−/−^ mice, which have a paucity of monocyte-derived macrophages, we identified candidate populations of Tim-4^+^ long-lived macrophages in both the LP (CD163^−^) and S/M (CD163^+^). Employing markers of longevity (Tim-4), maturity (CD4), and tissue localization (CD163) in *Cx3cr1*^creER^*R26-yfp* mice, we found that, up to 32 wk after tamoxifen treatment, macrophages expressing YFP were still present in both the LP and S/M. While the S/M macrophages were the longest-lived, a substantial proportion of LP macrophages were still expressing YFP at 32 wk. Thus, in contrast to the prevailing paradigm, macrophages in the tonically inflamed environment of the LP can persist for long periods of time. This finding is in contrast with a recent paper showing long-lived RTMs only in the S/M (De Schepper et al., 2018). We cannot currently explain this difference, as the mouse model and method used to label and detect long-lived RTMs were the same. As more groups become aware of, and study, these long-lived intestinal RTMs, a consensus on their location should emerge.

In addition to demonstrating the presence of long-lived Tim-4^+^ macrophages in the LP, we also identified CD163 as an unappreciated marker in the small intestine of macrophages restricted to the S/M (Fig. 1). CD163 is a scavenger receptor that acts by endocytosing hemoglobin–haptoglobin complexes (Kristiansen et al., 2001). Its expression has been associated with perivascular macrophages in mice, including those in the brain (Kim et al., 2006) as well as tumor-associated macrophages (Etzerodt et al., 2019). Whether S/M CD163^+^ macrophages in the gut are localized with blood vessels is not yet understood, but the identification of CD163 as a marker to distinguish between LP and S/M macrophages in the small intestine will provide new opportunities for selective targeting. For example, employing *Cd163*^cre^ animals (Etzerodt et al., 2019) to target S/M macrophages, with the caveat that vascular macrophages in other organs will be impacted.

Mapping of our findings of long-lived LP and S/M macrophages onto human macrophage populations remains to be undertaken. While Tim-4 itself is unlikely to be a conserved marker for human long-lived gut macrophages, based on its lack of expression in recent datasets (Domanska et al., 2022; Fenton et al., 2021, *Preprint*; Martin et al., 2019), the overall signatures observed may be more useful. Similarly, CD163 is reported to be expressed by macrophages within the villi of the human intestine (Bain et al., 2013), limiting the utility of this marker to identify regionally restricted RTMs to murine models. Aligning our data to human single-cell datasets may allow for potential alternative and conserved markers to be identified, such as the recently identified FOLR2, which was found to be expressed in submucosal and muscularis macrophages in human colonic samples (Domanska et al., 2022) and also identified to be a conserved marker of self-sustaining macrophage populations across tissues and species (Dick et al., 2022). In line with these reports, *Folr2* was identified as an upregulated DEG in cluster 1 (CD163^+^ macrophages) in our bulk RNA-seq data (Table S1).

Irrespective of the precise conserved factors between mice and humans, identification of long-lived Tim-4^+^ macrophages in both the LP and S/M provided us with an opportunity to understand how the gut-associated signal TGF-β acts across these two compartments. It is well established that global loss of TGF-β1 signalling causes multiorgan inflammation, including in the stomach and colon (Kulkarni et al., 1993; Shull et al., 1992), and total intestinal RTMs have been shown to require TGF-β signalling for their organ-specific differentiation (Schridde et al., 2017). However, studies looking at the effect of disrupting TGF-β signalling, specifically in myeloid cells, on the intestine have produced conflicting results (Bain et al., 2017; Ihara et al., 2016; Li et al., 2013; Ramalingam et al., 2012; Rani et al., 2011; Sanjabi and Flavell, 2010), and none have assessed how TGF-β signalling influences intestinal RTMs in different sub-tissular compartments or RTMs with different longevities. Due to the lack of tools available to target intestinal RTMs and specific subsets, we generated a *Timd4*^cre^ mouse, which allowed us to target Tim-4^+^ macrophages.

Using this new transgenic mouse, we identified differential effects of TGF-β signalling on two similarly long-lived Tim-4^+^ macrophage subsets, within different sub-tissular regions of the small intestine. TGF-β signalling was found to regulate CD163 expression and iNOS production by long-lived Tim-4^+^ macrophages in the LP, with loss of TGF-β signalling in these cells resulting in aberrant expression of these phenotypic and functional markers in long-lived LP macrophages. The consequences of the presence of dysregulated long-lived macrophages in the LP remain to be explored, but a predisposition to inflammatory disease is a possibility and may provide a mechanistic link between disrupted TGF-β signalling and inflammatory bowel disease (IBD) (Marafini et al., 2013).

When taken together, our data suggest two very distinct pathways for macrophage development in the small intestine, depending on sub-tissular location. Movement of monocytes into the LP is associated with a sequential acquisition of an LP macrophage-signature associated with length of time present in the tissue and relating to CD4 and Tim-4 expression (Fig. 1 G and Fig. 2 B). In the longest-lived LP macrophages, ongoing exposure to TGF-β leads to a hyporesponsive phenotype following exposure to IFN-γ/LPS (Fig. 4 C). Contrasting this, macrophages in the S/M are predicted to more rapidly adopt a transcriptional profile that does not further change with time spent in the tissue, as determined by pseudotime analysis and upregulation of CD4 and Tim-4 (Fig. 1 G and Fig. 2 B) and is associated with maintained responsiveness to IFN-γ/LPS stimulation (Fig. 4 C). Whether these alternative pathways for macrophage development in different sub-tissular location are dependent on the differences in TGF-β1 expression that we identify here, and further assessment of the other local environmental factors determining which pathway a monocyte takes upon entry into the intestine is still required. Interestingly, regionally specialized LepR^+^ fibroblasts that specifically contribute to the maintenance of CD163^+^ macrophages in the S/M have recently been described (Nonaka et al., 2025). Whether these regionally specialized fibroblasts contribute to the niche-dependent regulation of macrophage identity in ways other than through the production of growth factors should be investigated.

While CD163 is a useful marker to distinguish between LP and S/M macrophages in the small intestine, its utility in the colon is lessened by the expression of CD163 on LP macrophages. However, in agreement with other reports (Hegarty et al., 2026; Kang et al., 2020), we observed that CD163 expression by colonic LP macrophages identifies those in the lower crypt region, closer to the serosa, as well as S/M macrophages. We exploited the lower level of CD163 expression on macrophages in the upper crypt region to assess the effect of perturbing TGF-β1 signalling on CD163 expression by these regionally restricted macrophages. While we observed no increase in CD163 expression in the upper crypt macrophages of *Cx3cr1*^creER^*Tgfb1*^fl/fl^ mice, where *Tgfb1* was temporally deleted from all intestinal, CD163 expression was increased on upper-crypt macrophages from *Timd4*^cre^*Tgfbr2*^fl/fl^ mice, in which TGF-β signalling was constitutively perturbed. These results, combined with those from the small intestine, confirm regionally restricted effects of TGF-β signalling on intestinal macrophage identity and suggest that TGF-β availability and/or dependency differs across macrophages at different tissue sites in the intestine and potentially during different stages of development.

Overall, our new understanding of distinct long-lived macrophages will open up new routes for researching intestinal macrophage populations and inform understanding of signalling pathways that could be targeted for the treatment of intestinal inflammatory diseases.

## Materials and methods

### Mice

Male C57BL/6J (CD45.2) were purchased from Charles River Laboratories, strain #:000664 (experiments within the UK) or Janvier (experiment within Belgium) and housed in individually ventilated cages under specific pathogen−free (SPF) conditions. Germ-free C57BL/6 mice (founders from the Clean Mouse Facility, University of Bern, Bern, Switzerland) were bred and maintained in The University of Manchester gnotobiotic facility. The following mice, originally from The Jackson Laboratory, were bred in-house and housed in individually ventilated cages under SPF conditions: *Ccr2*^*−/−*^, strain #:004999 (Boring et al., 1997); *Cx3cr1*^creER^, strain #:020940 (Yona et al., 2013); *R26R-yfp*, strain #:006148 (Srinivas et al., 2001); *TbRII*, JAX stock #:012603 (Levéen et al., 2002); congenic CD45.1, strain #:002014 (Janowska-Wieczorek et al., 2001); *R26-tdrfp,* JAX stock #:038164 (Luche et al., 2007); C57BL/6J-*Tgfb1em2Lutzy/Mmjax*, JAX stock #:065809 (obtained from the Mutant Mouse Resource and Research Center [MMRRC] at The Jackson Laboratory, a National Institutes of Health–funded strain repository, donated to the MMRRC by Cat Lutz, PhD). C57BL/6J*-Ms4a3*^*Cre*^ mice (generated and provided by Dr Florent Ginhoux [Singapore Immunology Network, ASTAR] and Dr Zhaoyuan Liu [Shanghai Institute of Immunology] (Liu et al., 2019), crossed with *Rosa^t^*^*dT*^(Ai14) mice, strain #:007914 (Madisen et al., 2010) were maintained and used at the University of Edinburgh. *Timd4*^*Em1Uman*^ mice were generated as described below, by Antony D. Adamson, at The University of Manchester. *C57BL/6J.Timd4*^*Em1Uman*^ were bred with *TbRII* mice and referred to as *Timd4*^cre^*Tgfbr2*^fl/fl^ mice. *Cx3cr1*^creER^ were bred with *R26R-yfp* mice and with *R26-tdrfp* and *Tgfb1em2Lutzy/Mmjax* mice, respectively, referred to as *Cx3cr1*^creER^*R26-yfp* and *Cx3cr1*^creER^*Tgfb1*^fl/fl^ mice. Crossed *Cx3cr1*^creER^ and *Timd4*^cre^ Cre-lines were used as Cre heterozygous knockouts and Cre-negative littermate controls. All experiments were approved by The University of Manchester or Edinburgh Local Ethical Review Committee and were performed in accordance with the UK Home Office Animals (Scientific Procedures) Act 1986. Additionally, paraffin-embedded tissue samples from WT C57BL/6J treated with doxorubicin at the VIB-UGent Center for Inflammation Research were used. This work was approved by the VIB-UGent Center for Inflammation Research Ethical Committee and the University of Ghent Animal Ethics Committee.

### Generation of C57BL/6J.Timd4^Em1Uman^ mice

To express Cre recombinase under the control of *Timd4* regulatory regions but preserve Tim-4 expression, we used CRISPR-Cas9 to integrate Cre recombinase immediately downstream of the *Timd4* translation stat site, along with a T2A self-cleaving peptide, to ensure both genes/proteins are generated from the locus.

The sgRNA sequences, which target the Timd4 ATG (5′-GAT​CCT​ATC​AAA​ATG​TCC​AA-3′ and 5′-AGC​CCC​TTG​GAC​ATT​TTG​AT-3′) were purchased as full-length Alt-R sgRNA oligos (Integrated DNA Technologies) and resuspended in sterile, RNase-free injection buffer (Tris-HCl 1 mM, pH 7.5, and EDTA 0.1 mM). For our donor repair template, we used the Easi-CRISPR long-ssDNA strategy (Quadros et al., 2017) and generated a homology-flanked lssDNA donor using protocols described in Bennett et al. (2021).

For embryo microinjection, the annealed sgRNA was complexed with Cas9 protein (New England Biolabs) at room temperature for 10 min before addition of long ssDNA donor (final concentrations: sgRNA 20 ng/μl, Cas9 protein 20 ng/μl, lssDNA 10 ng/μl). CRISPR reagents were directly microinjected into the nuclei of C57BL/6J (Envigo) zygotes using standard protocols. Zygotes were cultured overnight, and the resulting two-cell embryos surgically implanted into the oviduct of day 0.5 postcoitum pseudopregnant mice.

Potential founder mice were screened by PCR, first using primers that flank the sgRNA sites (JG01_F 5′-GCC​ACC​ATG​AGA​AAA​GTG​CCT-3′; JG01_R 5′-TCC​CCA​AAC​ACC​CAA​ATC​CA-3′), which both identifies editing activity in the form of InDels from nonhomologous end joining repair and can also detect larger products, implying integration of the Cre-T2A through homology-directed repair. Secondary PCRs used the same primers in combination with Cre-specific primers (Cre_F 5′-GAT​CGC​TGC​CAG​GAT​ATA​CG-3′; Cre_R 5′-GTG​CCT​TCT​CTA​CAC​CTG​CG-3′). Candidate founders giving positive products in all three PCR reactions were further characterized by amplifying again with the JG01 F/R primers using high-fidelity Phusion polymerase (NEB); the larger product was gel extracted and subcloned into pCRblunt (Invitrogen) and Sanger sequenced with M13 forward and reverse primers. Alignment of the sequencing confirmed integration of the tag. A single founder was bred with WT C57BL/6J, and germline transmission was confirmed with the same array of assays.

### Paraffin-embedded tissues

Paraffin-embedded tissue samples were taken from mice subjected to the following treatments at The University of Manchester or the University of Edinburgh.

#### PLX treatment

Male and female WT mice on a C57BL/6 background were fed PLX3397 diet from 5 wk of age for a period of 2 wk to block the CSF1R and deplete macrophages. After 2 wk, mice were returned to normal chow and left for 7 wk to allow repopulation of macrophages from monocyte precursors.

#### Anti-CD3 treatment

Adult male mice on a C57BL/6 background were injected intraperitoneally with 20 μg anti-mouse anti-CD3e monoclonal antibody (clone 145-2C11; BioXcell) to induce intestinal inflammation. Mice were sacrificed 3 days after treatment, during the period in which inflammation is resolving.

#### Doxorubicin treatment

Adult male and female mice on a C57BL/6 background were injected intraperitoneally with 15 mg/kg doxorubicin (D-1515; Sigma-Aldrich) to induce intestinal inflammation. Mice were sacrificed 3 days after treatment, at the peak of bodyweight loss.

#### H. polygyrus infection

Adult male and female mice on a C57BL/6 background were infected with 200 L3 larvae by oral gavage. Mice were sacrificed on day 7 of infection, when the parasite is resident in the wall of the duodenum.

#### T. gondii infection

Adult mice on a C57BL/6 background were infected by oral gavage with 10^6^ PRU tachyzoites. Mice were sacrificed on day 10 of infection, a time point with visible intestinal inflammation.

### Tamoxifen treatment

Tamoxifen (Sigma-Aldrich) was dissolved in 10% ethanol and 90% corn oil to a concentration of 50 or 100 mg/ml. Males and females were dosed with 4 (*Cx3cr1*^creER^*Tgfb1*^fl/fl^) or 5 mg (*Cx3cr1*^creER^*R26-yfp*) by oral gavage for 5 consecutive days.

### FITC-dextran gut permeability assay

Male *Timd4*^cre^*Tgfbr2*^fl/fl^ (12–15 wk old) mice were fasted for 4 h prior to treatment. FITC-dextran (46944; Sigma-Aldrich), prepared at 100 mg/ml in PBS, was administered at 600 mg/kg body weight via oral gavage. Exactly 1 h after FITC-dextran administration, the mice were sacrificed, and cardiac blood was collected into heparin-coated Eppendorf tubes. Plasma was obtained by centrifugation at 3,000 RPM for 10 min at 4°C. Plasma samples were diluted 1:1 (vol/vol) in PBS, and their fluorescence was measured spectrophotometrically (Tecan Infinite 200 Pro) in black 96-well plates (excitation: 485 nm; emission: 525 nm). FITC-dextran concentrations were calculated using a standard curve prepared by diluting FITC-dextran in plasma collected from naïve mice, with concentrations ranging from 10.5 to 0.61 ug/ml.

### Generation of shielded chimeras

Male CD45.2^+^ host mice aged 6–8 wk were anaesthetized by intraperitoneal administration of ketamine (80 mg/kg; Vetoquinol) and xylazine (8 mg/kg; Bayer). Anesthetized mice were positioned beneath a lead sheet shielding the lower two-thirds of the body, including the intestine, from a split dose of irradiation (2 × 5.5 Gy). Mice therefore received partial body irradiation with only the head, thorax, and forelimbs left exposed. After recovery from anesthesia, mice were reconstituted by intravenous injection with 2 × 10^6^ CD90.2^+^ T cell–depleted donor BM cells from congenic CD45.1^+^ WT donor animals. T cells were depleted using CD90.2 microbeads (Miltenyi Biotec). Mice were maintained on 0.03% enrofloxacin in drinking water for up to 1 wk before and for 2 wk after irradiation and then were housed in autoclaved cages with sterile water, diet, and bedding. Reconstitution was allowed to occur for 24 wk before analysis.

### Tissue preparation and cell isolation

#### Intestine preparations

Cells were isolated as previously described (Shaw et al., 2018). In brief, after dissection of the intestine (and Peyer’s patches removed from the length of the small intestine), tissues were cut longitudinally and washed thoroughly with PBS on ice. Subsequently, to remove intestinal epithelial cells and leukocytes, intestines were cut into segments (2–3 cm) and incubated in prewarmed media (RPMI 1640) supplemented with penicillin and streptomycin, 3% FCS, 20 mM HEPES, 100 U/ml polymyxin B (Sigma-Aldrich), 5 mM EDTA, and 1 mM freshly thawed dithiothreitol for 15 min at 37°C with agitation. After incubation, intestinal segments were repeatedly shaken in fresh serum-free media with 2 mM EDTA and 20 mM HEPES to ensure optimal dissociation of intestinal epithelial cells and leukocytes. Remaining tissue (LP and muscularis) was minced and digested at 37°C for 30 min with continuous stirring in serum-free RPMI containing 20 mM HEPES, 0.1 mg/ml liberase TL (Roche), and 0.5 mg/ml DNase. Digested tissue was passed sequentially through a 70-µm and 40-µm cell strainer, and after pelleting, it was resuspended in RPMI 1640 supplemented with 2 mM L-glut, 1× nonessential amino acids (NEAA), 1 mM sodium pyruvate, 20 mM HEPES, 1× penicillin and streptomycin, and 10% FCS until staining.

#### Blood

Blood was collected into EDTA-coated syringes from sacrificed mice. Suspensions were washed and resuspended in ACK lysing buffer (Gibco) for 3 min on ice, twice. Suspensions were then washed and resuspended in RPMI 1640 supplemented with 2 mM L-glut, 1× NEAA, 1 mM sodium pyruvate, 20 mM HEPES, 1× penicillin and streptomycin, and 10% FCS until staining.

#### Bone marrow

Bone marrow was collected from hind-leg femurs, cut at one end, and placed inverted into collection tubes and pulsed in a microfuge. Cells were passed through a 70-µm filter and resuspended in ACK lysing buffer (Gibco) for 3 min on ice. Suspensions were then washed and resuspended in RPMI 1640 supplemented with 2 mM L-glut, 1× NEAA, 1 mM sodium pyruvate, 20 mM HEPES, 1× penicillin and streptomycin, and 10% FCS until staining.

#### Peritoneal exudate cells

Peritoneal cavity exudate was collected by injecting 8 ml PBS into the peritoneal cavity. The resulting cell suspension was then reclaimed, washed, and resuspended in RPMI 1640 supplemented with 2 mM L-glut, 1× NEAA, 1 mM sodium pyruvate, 20 mM HEPES, 1× penicillin and streptomycin, and 10% FCS until staining.

#### Liver

Liver tissue finely minced in pre-warmed digest media (RPMI 1640), supplemented with 20 mM HEPES, 1 mg/ml collagenase IV (Gibco), and 10 μg/ml Dnase (Sigma-Aldrich) and incubated for 20 min at 37°C with continuous stirring. After incubation, the digested livers were passed sequentially through a 70-µm and 40-µm cell strainer and, after pelleting, were resuspended in RPMI 1640 supplemented with 2 mM L-glut, 1× NEAA, 1 mM sodium pyruvate, 20 mM HEPES, 1× penicillin and streptomycin, and 10% FCS until staining.

### *In vitro* assays

For stimulations, whole small intestine, prepared as above, was pelleted and resuspended in 5 ml of NycoPrep (Axis Shield) or OptiPrep (STEMCELL Technologies), prepared according to the manufacturer’s directions. 2 ml of FCS-free RPMI was carefully layered on top, and the preparation was centrifuged at 800 *g* for 15 min at room temperature with the brake set to “low.” Mononuclear phagocytes at the interface were collected into a fresh tube, counted, and 1 × 10^6^ cells were seeded for stimulations.

#### Pro-IL-1β and TNF-α induction

1 × 10^6^ cells were incubated in RPMI 1640 supplemented with 2 mM L-glut, 1× NEAA, 1 mM sodium pyruvate, 20 mM HEPES, 1× penicillin and streptomycin, and 10% FCS in a 96-well “U”-bottomed plate and stimulated with 1 μg/ml LPS-EB Ultrapure (InvivoGen) and Brefeldin A (eBioScience). After 3 h, cells were washed and stained with antibodies for assessment by flow cytometry.

#### iNOS induction

1 × 10^6^ cells were seeded in a non-tissue culture treated 24-well plate in RPMI 1640 supplemented with 2 mM L-glut, 1× NEAA, 1 mM sodium pyruvate, 20 mM HEPES, 1× penicillin and streptomycin, 50 mg/ml gentamicin, and 10% FCS. Cells were incubated at 37 °C and 5% CO_2_ for 1 h to settle and then stimulated with 20 ng/ml recombinant IFN-γ (BioLegend), 100 ng/ml LPS-EB Ultrapure (InvivoGen), and 20 ng/ml recombinant murine M-CSF (PeproTech) overnight. The next day, cells were collected from the supernatant and detached from the plate using ACCUTASE (STEMCELL Technologies) for assessment by flow cytometry.

#### Phagocytosis assay

1 × 10^6^ cells from whole small intestine, prepared as above, were incubated in RPMI 1640 supplemented with 2 mM L-glut, 1× NEAA, 1 mM sodium pyruvate, 20 mM HEPES, 1× penicillin and streptomycin, and 10% FCS in a 96-well U-bottomed plate for 30 min before the addition of 10 ml of pHrodo Red *S. aureus* BioParticles Conjugate for Phagocytosis, prepared according to the manufacturer’s guidelines (Thermo Fisher Scientific). After 40 min, cells were washed and stained with antibodies for assessment by flow cytometry.

### Flow cytometry

Single-cell suspensions, prepared as described above, were washed with PBS and stained with the LIVE/DEAD Fixable blue or Near-IR Dead Cell Stain kit (Thermo Fisher Scientific) to exclude dead cells. Subsequently, cells were stained in the dark for 15 min at 4°C with fluorochrome-conjugated antibodies in PBS containing anti-CD16/CD32 (2.4G2; BioXcell or BioLegend). Cells were washed and, in some cases, immediately acquired live, or alternatively, cells were fixed in 2% paraformaldehyde (PFA) (Sigma-Aldrich) for 10 min at room temperature and resuspended in PBS for later acquisition. Cells were stained with CD4 (RM4-5), CD11b (M1/70), CD11c (N418), CD45 (30F11), CD45.1 (A20), CD45.2 (104), CD64 (X54-5/7.1), CD115 (AFS98), MHCII (I-A/I-E; M5/114.15.2), and Tim-4 (RMT4-54) from BioLegend as well as CD163 (TNKUPJ) and Ly6C (HK1.4) from Thermo Fisher Scientific. The lineage antibody cocktail for excluding lymphocytes and granulocytes included Siglec F (E50-2440) from BD and TCRβ (H57-597), B220 (RA3-6B2), and Ly6G (1A8) from BioLegend. For intracellular antibody staining, fixed cells were permeabilized with Permeabilization Buffer (Thermo Fisher Scientific) and stained with CD63 (NVG-2) and CD206 (C068C2) from BioLegend and pro-IL-1β (NJTEN3), TNF-α (MP6-XT22), and iNOS (CXNFT) from Thermo Fisher Scientific. Cell acquisition was performed on an LSRFortessa running FACSDIVA software (BD) or SONY ID7000 (SONY). For each intestinal sample, typically 10,000–20,000 macrophages were collected. Data were analyzed using FlowJo software (TreeStar).

#### Fluorescence activated cell sorting

Single-cell suspensions for small intestine were prepared as above. Before FACS, on a FACSAria Fusion (BD), isolated cells were suspended in RPMI supplemented with 2% FCS and 2 mM EDTA. Sorted cells were collected in RLT buffer (QIAGEN) supplemented with 2-mercaptoethanol (Sigma-Aldrich) and stored on dry ice before storage at −80°C for subsequent RNA extraction.

#### Bulk RNA-seq

RNA was extracted from 25,000–50,000 cells using an RNeasy micro kit (QIAGEN), following the manufacturer’s instructions. RNA quality was checked using a LabChip GX RNA pico kit (Perkin Elmer) or Bioanalyzer RNA 6000 pico kit (Agilent), and samples were found to have RNA integrity number values above seven. RNA-seq libraries were prepared by Edinburgh Genomics using a SMART-Seq version 4 PLUS RNA Kit (Takara Bio USA) with nine cycles of amplification. Library cDNA quality was assessed by HSDNA kit on a 2100 Bioanalyzer (Agilent). 16–24 million reads were obtained from each sample using a NovaSeq 6000 platform (Illumina). Reads were filtered with Trimmomatic (version 0.36) (Bolger et al., 2014). Filtered fastq files were aligned to the mouse GENCODE genome (GRCm38.p5) using STAR (version 2.5.3) (Dobin et al., 2013). Filtered reads were then sorted, compressed, and unaligned reads were removed using Samtools (version 1.3) (Li, 2011; Li et al., 2009). Aligned reads were then counted, normalized, and compared, respectively, using the Cuffquant, Cuffnorm, and Cuffdiff function of Cufflinks (version 2.2.2) (Roberts et al., 2011; Trapnell et al., 2013; Trapnell et al., 2010). Heatmaps were visualized using Morpheus (https://software.broadinstitute.org/morpheus) (Morpheus, 2025). Genes were clustered using one minus Pearson correlation k-means clustering. GO and pathway analyses were conducted with PANTHER (http://pantherdb.org). Bulk RNA-seq data were deposited in the Gene Expression Omnibus public database under accession no. GSE232645.

### scRNA-seq

#### Single-cell isolation and sequencing

Single-cell suspensions of three pooled small intestines of male C57BL/6J or four pooled small intestines of male *Ccr2*^−/−^ mice were FACS purified for live CD45^+^ Ly6C^−^ Ly6g^−^ TCRb^−^ CD3^–^ B220^−^ Siglec F^−^ CD11b^+^ MHC II^+^ CD64^+^ macrophages. Gene expression libraries were prepared from single cells using the Chromium Controller and Single Cell 3ʹ Reagent Kit version 2 (10x Genomics) according to the manufacturer’s protocol to generate single-cell gel bead-in-emulsion. scRNA-seq libraries were prepared using GemCode Single-Cell 3ʹGel Bead and Library Kit (10x Genomics) according to the manufacturer’s instructions. Indexed sequencing libraries were generated using the reagents in the GemCode Single-Cell 3ʹ Library Kit and sequenced using the Illumina NextSeq500 platform. Sequencing was performed at the VIB Nucleomics Core (Leuven, Belgium) (C57BL/6J) or at The University of Manchester Genomic Technologies Core Facility (Manchester, UK) (*Ccr2*^−/−^). The .bcl sequence data were processed for quality control purposes using bcl2fastq software (version 2.20.0.422), and the resulting .fastq files were assessed using FastQC (version 0.11.3), FastqScreen (version 0.9.2), and FastqStrand (version 0.0.5) before preprocessing with the Cell Ranger pipeline.

### Data processing

Cells with >10% mitochondrial reads were filtered out. The remaining cells were normalized, integrated (using IntegrateData), and analyzed with Seurat (version 5.3.0). Gene set module enrichment is presented as a proportion of the transcript count of the genes comprising the gene set module within the total transcript count of each cell and visualized using FeaturePlot (Seurat version 5.3.0). Trajectory analysis was performed using Monocle 3 version 1.3.7 (Trapnell et al., 2014), with cluster 0 as the first root. scRNAseq data were deposited in the Gene Expression Omnibus public database under accession no. GSE234018.

#### Public dataset comparisons

For comparison of scRNA-seq data with LP and serosal macrophage and monocyte signatures from the ImmGen database, the ImmGen V1 microarray dataset was used. Serosal cells were identified as propidium iodide^−^ CD45^+^ MHC II+ CD11c^lo^ CD103^−^ CD11b^+^. LP cells were identified as propidium iodide^–^ CD45^+^ MHC II^+^ CD11c^hi^ CD103^–^ CD11b^+^ (Heng and Painter, 2008). Genes that were expressed more than twofold in MF_103-11b+_SI relative to MF_11cloSer_SI were selected as the LP gene signature, and genes that were expressed more than twofold in MF_11cloSer_SI relative to MF_103-11b+_SI as the Serosa gene signature. Finally, genes that were expressed more than twofold in Mo_6C+II-_Bl relative the mean of MF_103-11b+_SI and MF_11cloSer_SI macrophages were selected as the classical monocyte gene signature. Violin plots show the sum of the transcripts of the genes within the gene signatures as a ratio of the total transcripts (transcript per million of gene set of interest/transcript per million of gene set from ImmGen datasets).

### Histology and immunofluorescence microscopy

#### Sample preparation and storage

Intestines were flushed with PBS, followed by 4% PFA. Short intestinal tissue sections and Swiss rolls were drop fixed in 4% PFA for 16–24 h at 4°C and subsequently embedded and stored in paraffin blocks or drop fixed in 4% PFA containing 30% sucrose for 3–4 h at 4°C and subsequently snap frozen in OCT in isopentane on dry ice and stored at −80°C.

#### Histology

Paraffin sections of 5 μm were subjected to normal deparaffinization and hydration and stained with hematoxylin solution, Gill no. 3 (Sigma-Aldrich) and eosin Y (Sigma-Aldrich), mounted with DPX (VWR), and coverslipped.

#### Immunofluorescence

Paraffin sections of 5–8 μm were subjected to normal deparaffinization and hydration. Antigen retrieval was carried out using Tris-EDTA, pH 9. Tissues were blocked for endogenous biotin using an avidin biotin blocking kit (BioLegend), according to the manufacturer’s instructions, and incubated overnight at 4°C with one of the following primary antibodies: rat anti-Tim-4 (RMT4-54; BioLegend), goat anti-Tim-4 (AF2826; bio-techne), or rabbit anti-CD163 (ab182422; Abcam), diluted in TBS supplemented with 1% BSA + 0.3% Triton X. The following day, tissues were treated with 0.03–0.3% H_2_O_2_ to block endogenous peroxidase before incubation for 1 h at room temperature with biotin-conjugated antibodies against the species of the primary antibody, biotin anti-rat and biotin anti-rabbit. Tissues were subsequently incubated for 1 h at room temperature with HRP-conjugated streptavidin, followed by Tyramide 555, prepared according to the manufacturer’s instructions (Thermo Fisher Scientific). Tissues stained with CD163 were then subject to antigen retrieval using sodium citrate pH 6 and incubated overnight at 4°C with rabbit anti-IBA1 (ab178846; Abcam), followed by anti-rabbit AF488 secondary antibody or with rabbit anti-IBA1 AF647 (ab225261; Abcam). Tissues stained with Tim-4 were incubated overnight at 4°C with rabbit anti-IBA1 (ab178846; Abcam), followed by anti-rabbit AF488 secondary antibody or with rabbit anti-IBA1 AF647 (ab225261; Abcam). Finally, tissues were counterstained with DAPI, mounted in prolong diamond mountant (Thermo Fisher Scientific) and coverslipped.

For detection of endogenous YFP fluorescence, frozen sections of 10 μm were incubated for 1 h with AF647 anti-Iba1 antibody (ab225261; Abcam). Tissues were then counterstained with DAPI, mounted in prolong diamond mountant (Thermo Fisher Scientific), and coverslipped.

Images were collected on a Zeiss Axioimager.D2 upright microscope using a 10× air objective and captured using a Coolsnap HQ2 camera (Photometrics) through Micromanager software version 1.4.23 or on a Nikon Ti2 Eclipse widefield live imaging microscope on a 20× air objective and captured using a Photometrics Prime 95B CMOS camera. Endogenous YFP was captured using a YFP filter (ex 510/25 nm, em 535/30 nm) in OCT-embedded sections of small and large intestine from *Cx3cr1*^*creER*^*:R26-yfp* mice. Final images for publication were processed using ImageJ to create merged images and montages (Schneider et al., 2012).

For quantification of CD163^+^ cells, the area of staining for CD163/mm^2^ was calculated and normalized to the DAPI signal from five images per section. Number of cells per mm^2^ were counted using QuPath (Bankhead et al., 2017). Initially, all the images were loaded onto QuPath, and the CD163^+^ cells were segmented with Cellpose “cyto3” (Stringer and Pachitariu, 2025) plugin with a pixel size = 0.4 μm, median cell diameter = 20–25 μm, and flow threshold = 0.6. An object classifier was then created detecting CD163-positive cells on a full image annotation. For quantification of TGF-β1 and SMAD3, the nuclei were segmented based on DAPI using “stardist” (Schmidt et al., 2018) on a full image annotation. An object classifier was then created and trained using four random images for detecting TGF-β1 or SMAD3 with IBA1 using the Random Trees Classifier to detect positive cells for TFGβ1, SMAD3, and IBA1. After segmentation and classifications, ROIs were made to calculate cells in the LP and S/M. The above steps were batched and applied to all images and exported as a CSV file for further analysis. For making the spatial distribution plot of CD163 and TGF-β1 signal, ROIs were made around a complete villus or an entire leaf like section of the colon. Cellpose cyto3 or stardist was used to segment all the nuclei in these ROIs, and the distance of the cells was calculated from the tip was calculated. The single-cell level information was exported as a CSV. The spatial plots were made using ggplot in Rstudio (version 4.5.0) using the geom_smooth function using a locally estimated scatterplot smoothing of the signal intensity of CD163 or TGF-β1. The cell distribution map of the small and colon was plotted as a dot plot using the x and y coordinates of the centroids of all segmented cells.

### Statistical analyses

Statistical analyses were performed using Prism 9 (GraphPad Software). A Shapiro–Wilk test was used to test for normality. For parametric data, two experimental groups were compared using a Student’s *t* test, for paired data, or a Student’s *t* test with Welch’s correction for unpaired data. For nonparametric data, two experimental groups were compared using a Mann–Whitney test. Where more than two groups were compared, a one-way ANOVA with Tukey’s multiple comparison test, or a Brown–Forsythe and Welch test, was used for parametric data, and a Kruskal–Wallis test with Dunn’s multiple comparison test was used for nonparametric data. Significance was set at P ≤ 0.05.

### Online supplemental material

Fig. S1 contains supporting data for Fig. 1, including the gating strategy for sorting macrophages from which scRNA-seq datasets were generated and immunofluorescence images of CD163^+^ macrophages in small intestine sections from mice subject to a variety of infectious, inflammatory, or non-homeostatic conditions. Fig. S2 contains supporting data for Fig. 2, including a projection of our bulk RNA-seq data onto our scRNA-seq data. Fig. S3 contains supporting data for Figs. 3 and 4, including data from fate-mapping and bone marrow chimera models demonstrating the monocyte contribution to small intestinal macrophage subsets and biological and technical controls for the *ex vivo* stimulation assay. Fig. S4 contains supporting data for Fig. 5, including the expression of genes from the TGF-β receptor signalling pathway GO term (GO-0007179) in small intestinal macrophage by scRNA-seq. Fig. S5 contains supporting data for Figs. 6 and 7, including quantification of the Tim-4^+^ macrophage population in *Timd4*^cre^*Tgfbr2*^fl/fl^, *Cx3cr1*^creER^*Tgfb1*^fl/fl^ mice and their respective littermate controls. Table S1 contains all 3,206 DEGs identified in small intestinal macrophage populations sorted for bulk RNAseq based on their expression of CD4, Tim-4, and CD163.

## Supplementary Material

Table S1 shows all 3,206 DEGs identified in small intestinal macrophage populations sorted for bulk RNA-seq based on their expression of CD4, Tim-4, and CD163.

## Data Availability

scRNA-seqdata were deposited in the Gene Expression Omnibus public database under accession no. GSE234018. Bulk RNA-seq data were deposited in the Gene Expression Omnibus public database under accession no. GSE232645. All other raw data are available from the corresponding author upon reasonable. For the purpose of open access, the author has applied a Creative Commons Attribution (CC BY) licence to any Author Accepted Manuscript version arising from this submission.
